# Atomically Dispersed Copper Sites in a Metal–Organic Framework for Reduction of Nitrogen Dioxide

**DOI:** 10.1021/jacs.1c03036

**Published:** 2021-07-19

**Authors:** Yujie Ma, Xue Han, Shaojun Xu, Zi Wang, Weiyao Li, Ivan da Silva, Sarayute Chansai, Daniel Lee, Yichao Zou, Marek Nikiel, Pascal Manuel, Alena M. Sheveleva, Floriana Tuna, Eric J. L. McInnes, Yongqiang Cheng, Svemir Rudić, Anibal J. Ramirez-Cuesta, Sarah J. Haigh, Christopher Hardacre, Martin Schröder, Sihai Yang

**Affiliations:** † Department of Chemistry, University of Manchester, Manchester M13 9PL, United Kingdom; ‡ UK Catalysis Hub, Research Complex at Harwell, Rutherford Appleton Laboratory, Harwell OX11 0FA, United Kingdom; § ISIS Facility, STFC Rutherford Appleton Laboratory, Chilton, Oxfordshire OX11 0QX, United Kingdom; ∥ Department of Chemical Engineering and Analytical Science, University of Manchester, Manchester M13 9PL, United Kingdom; ⊥ Department of Materials, University of Manchester, Manchester M13 9PL, United Kingdom; # Photon Science Institute, University of Manchester, Oxford Road, Manchester M13 9PL, United Kingdom; ∇ Neutron Scattering Division, Neutron Sciences Directorate, Oak Ridge National Laboratory, Oak Ridge, Tennessee 37831, United States; ○ School of Chemistry, Cardiff University, Main Building, Park Place, Cardiff CF10 3AT, United Kingdom

## Abstract

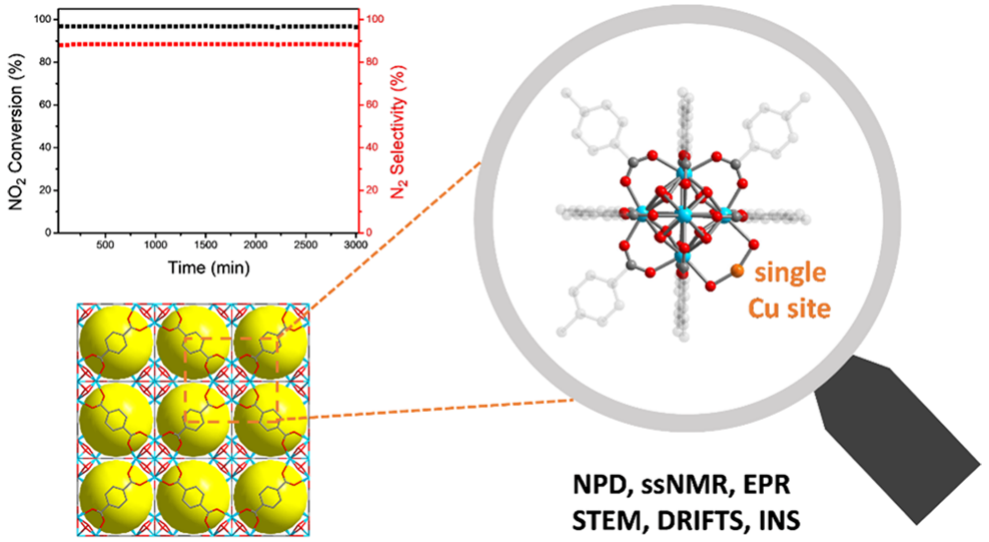

Metal–organic framework (MOF) materials provide an excellent platform to fabricate single-atom catalysts due to their structural diversity, intrinsic porosity, and designable functionality. However, the unambiguous identification of atomically dispersed metal sites and the elucidation of their role in catalysis are challenging due to limited methods of characterization and lack of direct structural information. Here, we report a comprehensive investigation of the structure and the role of atomically dispersed copper sites in UiO-66 for the catalytic reduction of NO_2_ at ambient temperature. The atomic dispersion of copper sites on UiO-66 is confirmed by high-angle annular dark-field scanning transmission electron microscopy, electron paramagnetic resonance spectroscopy, and inelastic neutron scattering, and their location is identified by neutron powder diffraction and solid-state nuclear magnetic resonance spectroscopy. The Cu/UiO-66 catalyst exhibits superior catalytic performance for the reduction of NO_2_ at 25 °C without the use of reductants. A selectivity of 88% for the formation of N_2_ at a 97% conversion of NO_2_ with a lifetime of >50 h and an unprecedented turnover frequency of 6.1 h^–1^ is achieved under nonthermal plasma activation. *In situ* and *operando* infrared, solid-state NMR, and EPR spectroscopy reveal the critical role of copper sites in the adsorption and activation of NO_2_ molecules, with the formation of {Cu(I)···NO} and {Cu···NO_2_} adducts promoting the conversion of NO_2_ to N_2_. This study will inspire the further design and study of new efficient single-atom catalysts for NO_2_ abatement *via* detailed unravelling of their role in catalysis.

## Introduction

Emerging single-atom catalysts show superior selectivity and activity in a variety of catalytic systems due to their unique electronic properties, low-coordination (or unsaturated) metal sites, and high atom efficiency.^1−4^ Metal–organic framework (MOF) materials are a class of crystalline porous materials that are ideal platforms for the fabrication of single-atom catalysts due to their uniform and well-defined structure, ultrahigh porosity, and designable functionality and pore sizes.^5−8^ Functional groups, such as hydroxyl bridges, and intrinsic defect sites in MOFs can enable the immobilization of atomically dispersed metal sites on the pore interior.

MOF-based single-atom catalysts have shown great promise in a number of catalytic reactions, yet the precise identification of these single-atom metal sites and their roles in catalysis remain elusive.^9−15^ As a result, there is a strong motivation to develop and apply cutting-edge experimental techniques to the investigation of local structures of the single metal sites, as well as the elucidation of their roles in catalytic process, thus unravelling the catalytic pathway and mechanism. Recently, a MOF-based single-atom catalyst, Cu/UiO-66, has been prepared by anchoring single-atom Cu sites to the defect sites of UiO-66 and used for catalytic CO oxidation and removal.^16^ Herein, we conclusively identify the location of the Cu sites in Cu/UiO-66, as well as their role in the catalytic reduction of NO_2_ to N_2_, an important process for NO_*x*_ abatement. The local structure of structural defects and atomically dispersed Cu sites within the framework and their role in promoting the activation of substrates are elucidated by *in situ* electron paramagnetic resonance (EPR) spectroscopy, neutron powder diffraction (NPD), inelastic neutron scattering (INS), solid-state nuclear magnetic resonance (NMR) spectroscopy, diffuse reflectance infrared Fourier transform spectroscopy (DRIFTS), high-angle annular dark-field scanning transmission electron microscopy (HAADF-STEM), and density functional theory (DFT) calculations. In addition to Cu/UiO-66, the catalytic performance of the −NH_2_ functionalized analogue of UiO-66 (UiO-66-NH_2_) has also been studied to gain insight into the potential activity of −NH_2_ groups in the reduction of NO_2_. Of all the catalysts studied herein, Cu/UiO-66 exhibits superior catalytic performance for the reduction of NO_2_ to N_2_ at room temperature without using any reducing agent under the activation of nonthermal plasma (NTP). Cu/UiO-66 shows a superior catalytic stability of >50 h in a continuous flow reaction, affording a N_2_ selectivity of 88% at a 97% conversion of NO_2_ with an unprecedented turnover frequency (TOF) of 6.1 h^–1^.

## Results and Discussion

### Synthesis and Characterization of Catalysts

**Figure 1:**
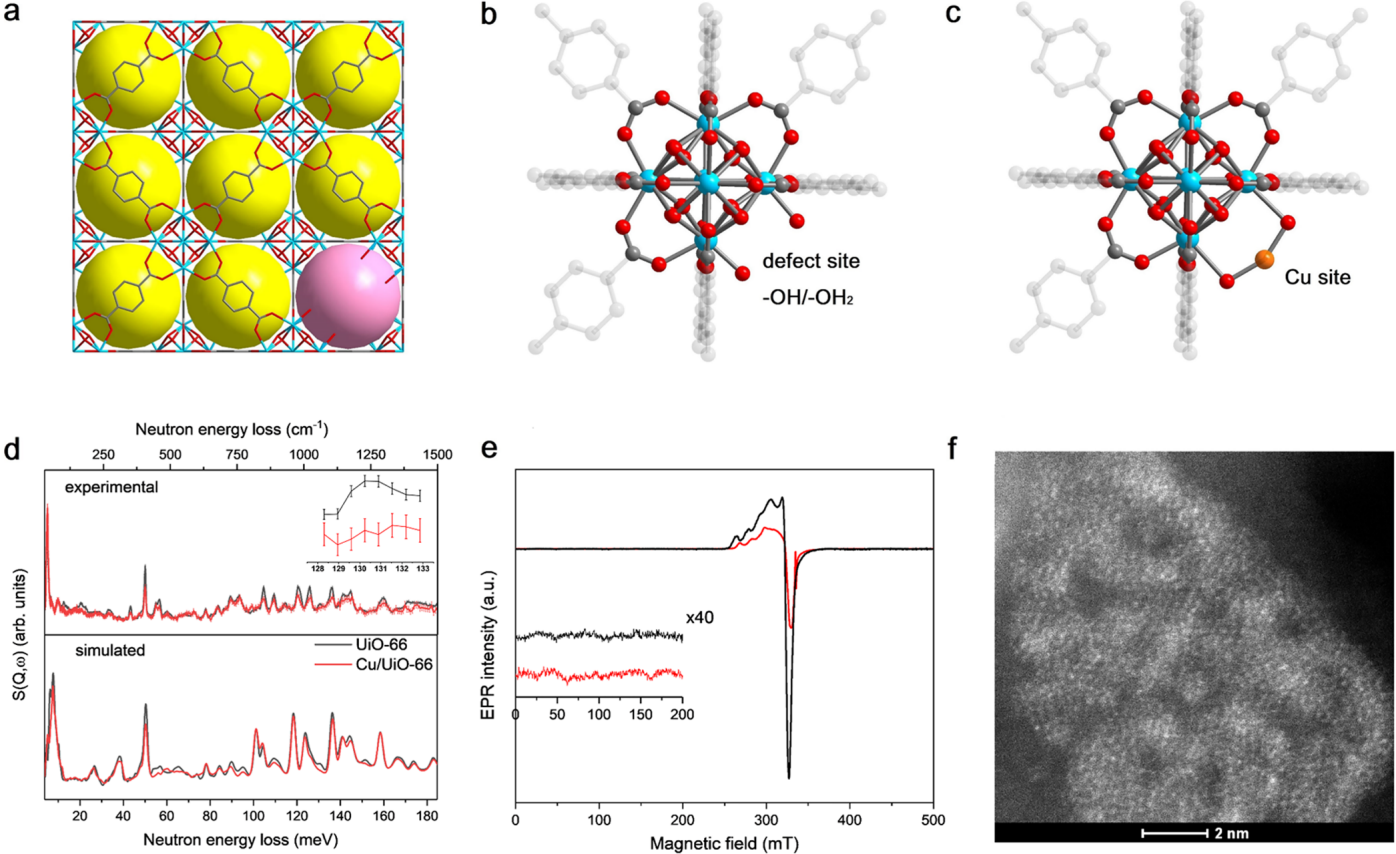
(a) Crystal structure of UiO-66 with a defect site. Yellow and pink spheres represent the cages without and with defect sites, respectively. (b) View of the {Zr_6_} cluster in UiO-66 with a defect site and (c) Cu/UiO-66 with a single-atom Cu site coordinated to −OH/–OH_2_ defect sites. These structures were derived from Rietveld refinements of the NPD data at 7 K (C, gray; O, red; Zr, blue; Cu, orange; H atoms are omitted for clarity). The bond distance of Cu–O is 1.88(3) Å, and the bond angle of O–Cu–O is 152.2(1)°. (d) Comparison of the experimental and DFT calculated INS spectra of UiO-66 and Cu/UiO-66 (inset: magnification of the experimental INS features at around 130 meV). (e) X-band (9.4 GHz) EPR spectra of Cu/UiO-66 before (black) and after H_2_ reduction (red), at 6.4 K (inset: expansion of low-field region confirming the absence of characteristic signals for binuclear or aggregated Cu species with *S* ≥ 1). (f) HAADF-STEM image of Cu/UiO-66 after H_2_ reduction.

UiO-66 was synthesized according to a reported method (Section S1.1).^16^ UiO-66-NH_2_ was prepared using the amine-functionalized ligand (2-aminoterephthalic acid) following the same procedure used for synthesizing UiO-66 (Section S1.1). Powder X-ray diffraction (PXRD) patterns (Figure S1), N_2_ adsorption isotherms (Figure S2), scanning electron microscopy (SEM, Figure S3) images, and thermal gravimetric analysis (TGA, Figure S4) confirmed the successful preparation of UiO-66 and UiO-66-NH_2_. The presence of structural defects (missing ligand) in desolvated UiO-66 was confirmed by NPD (Figure 1a,b), which revealed approximately one missing ligand per {Zr_6_} cluster, giving a formula of [Zr_6_O_4_(OH)_4_(BDC)_5.35_(OH)_1.30_(OH_2_)_1.30_] (H_2_BDC ligand = benzene-1,4-dicarboxylic acid), in good agreement with previous reports.^16−18^

Cu/UiO-66 was prepared through the attachment of Cu(II) ions to the defect sites of UiO-66, followed by reduction by 5% H_2_ in Ar at 250 °C for 2 h.^16^ The PXRD pattern (Figure S1), N_2_ adsorption isotherms (Figure S2), and SEM images (Figure S3) of Cu/UiO-66 confirm the retention of crystallinity, porosity, and crystal morphology upon the attachment of Cu to UiO-66. Elemental analysis suggested a ratio of Cu/{Zr_6_} of ∼1.0, i.e., all defect sites have been filled by Cu sites.

### Studies of the Atomically Dispersed Cu Sites

The location of Cu sites within Cu/UiO-66 has been determined by the Rietveld refinement of NPD data collected at 7 K (Figure 1c). Cu is coordinated to the −OH/–OH_2_ defect sites of UiO-66 with the ratio of Cu/{Zr_6_} cluster being ∼1.0. The Cu–O distance [1.88(3) Å] is in excellent agreement with that (1.89–1.95 Å) obtained from DFT calculations.^16^ The binding of Cu to the −OH/–OH_2_ defect sites was confirmed further by a combination of inelastic neutron scattering (INS) and DFT calculations (Figure 1d). Upon the binding of Cu, the INS features at 57 and 130 meV, assigned to O–H bending modes, show notable reductions in intensity, confirming the coordination of Cu to these defect sites, and this was validated by DFT simulations of the INS spectra. The simulations tend to slightly underestimate the magnitude of the reduction in intensities of the small features at 100–160 meV, likely due to the overall complexity of the system. This represents the first study of the location of Cu sites within Cu/UiO-66 by diffraction techniques.

Following the determination of the location of the Cu sites, it was established that these were primarily atomically dispersed. Thus, the continuous-wave EPR spectra of Cu/UiO-66 before and after reduction under H_2_ both show the characteristics of monomeric Cu(II) sites (Figure 1e).^19^ No binuclear (*S* = 1) or aggregated (long-range magnetically coupled) Cu(II) species was observed by the X-band EPR spectra collected at low and room temperatures (Figure 1e and Figure S7),^20^ consistent with the presence of atomically dispersed Cu(II) sites in the framework. The EPR spectra of reduced Cu/UiO-66 show that the majority of the Cu(II) sites are reduced to diamagnetic Cu(I) (Figure S8), which is consistent with X-ray photoelectron spectroscopic (XPS) analysis (Figure S17). The high-angle annular dark-field scanning transmission electron microscopy (HAADF-STEM) of Cu/UiO-66 shows many bright dots, representing Cu and Zr sites (Figure 1f and Figure S9a).^21^ Although it is challenging to distinguish between Cu and Zr centers,^22^ the HAADF-STEM image confirms the absence of Cu/CuO_*x*_ nanoparticles. Energy dispersive X-ray spectroscopy (EDX) mapping also shows a uniform distribution of the Cu sites (Figure S9c,d). This was further validated by the ultraviolet–visible (UV–vis) absorption spectrum of Cu/UiO-66, where the characteristic absorption peak of Cu nanoparticles [surface plasmon resonance (SPR) band] in the region of 550–600 nm^23^ was not observed (Figure S10).

### Analysis of Structural Defects and the Environment of Cu by Solid-State NMR

**Figure 2:**
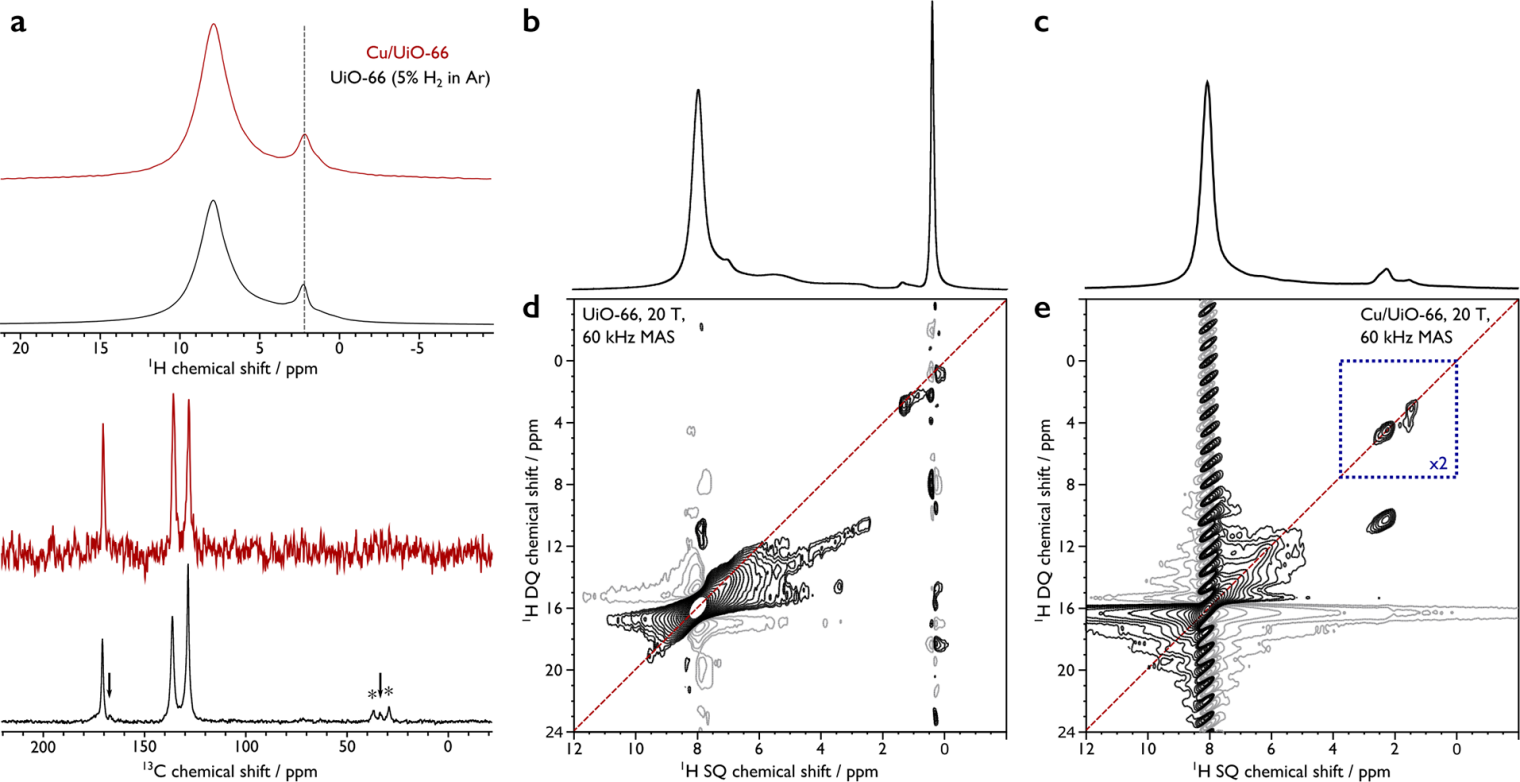
(a) ^1^H DEPTH (top) and {^1^H-}^13^C CP (bottom) MAS NMR spectra of H_2_-reduced UiO-66 (black) and Cu/UiO-66 (red). The spectra were recorded at 9.4 T using a MAS frequency of 10 kHz. The arrows and asterisks denote trace adsorbed DMF molecules and the spinning side bands, respectively. The dashed vertical gray line highlights the defect sites. ^1^H DEPTH MAS NMR spectra of (b) UiO-66 and (c) Cu/UiO-66. 2D ^1^H MAS NMR DQ-SQ homonuclear dipolar correlation spectra of (d) UiO-66 and (e) Cu/UiO-66, recorded using the S_3_ recoupling sequence; black and gray lines represent positive and negative contours, respectively, and the dashed red line highlights the DQ diagonal. The blue square in (e) represents an area where the minimum displayed contour level has been decreased by a factor of 2 (equivalent to doubled magnification). The spectra were recorded at 20 T using a MAS frequency of 60 kHz.

To gain further insights into the local environment of defects and Cu sites, solid-state NMR spectroscopy was employed as this has proven extremely valuable in the study of UiO-66 materials.^24,25^ Here, a quantitative ^1^H magic angle spinning (MAS) NMR spectrum of reduced UiO-66, obtained following the same treatment as Cu/UiO-66 (i.e., heating in 5% H_2_ in Ar at 250 °C for 2 h), shows that this treatment removes the H center from the μ^3^–OH species of the {Zr_6_} cluster (Figure 2a, top), which was previously observed by variable-temperature FTIR studies.^26^^1^H NMR signals from low-shifted species (δ{^1^H} < 2.5 ppm) account for ∼1/10th of the integral intensity compared to ^1^H from the ligands (δ {^1^H} ∼ 8 ppm) (Figure S11 and Table S3), whereas this intensity is expected to be 4 times larger for fully hydroxylated UiO-66. Therefore, these low-shifted species were assigned as −OH/–OH_2_ centers at defect sites. The ^1^H MAS NMR spectrum of Cu/UiO-66 (Figure 2a, top) is similar to that of reduced UiO-66, indicating that the majority of Cu(II) sites were reduced to Cu(I); a close proximity to paramagnetic Cu(II) ions would cause a substantial broadening and/or shifts of the NMR signals,^27^ and this was not observed. The similarity between the ^13^C MAS NMR spectra of reduced UiO-66 and Cu/UiO-66 (Figure 2a, bottom) demonstrates that the incorporation of Cu sites does not perturb the local ligand structure.

High-field (20 T) ^1^H NMR spectroscopy with ultrafast MAS was also employed as this provides superior resolution. The two-dimensional (2D) double-quantum single-quantum (DQ-SQ) ^1^H homonuclear dipolar correlation NMR spectrum (Figure 2d) for pristine UiO-66 shows the μ^3^–OH species at δ {^1^H} ∼ 0.4 ppm (Figure 2b and Figure S11). The remaining low-shifted protons at δ {^1^H} < 2.5 ppm display both diagonal autocorrelation peaks (between equivalent proton sites at δ_DQ_ {^1^H} ∼ 2.7 ppm) and off-diagonal correlation peaks (between inequivalent proton sites at δ_DQ_ {^1^H} ∼ 2.4 ppm). Accordingly, these proton environments can be assigned to defect sites (Figure 1b), where μ^1^–OH_2_ species (δ_SQ_ {^1^H} ∼ 1.3 ppm) will give an autocorrelation peak and nearby μ^1^–OH species (δ_SQ_{^1^H} ∼ 1.1 ppm) will be correlated to these (off-diagonal).

The high-resolution ^1^H MAS NMR spectrum of Cu/UiO-66 confirms the absence of μ^3^–OH species for this sample (Figure 2c). It is also evident that there are at least three distinct defect −OH/–OH_2_ environments at δ {^1^H} ∼ 2.4, 2.2, and 1.5 ppm. The 2D DQ-SQ ^1^H homonuclear dipolar correlation NMR spectrum of Cu/UiO-66 (Figure 2e) displays clear and intense autocorrelation peaks for two of the defect sites (at δ_SQ_ {^1^H} ∼ 2.4 and 2.2 ppm), indicating that they arise from OH_2_ environments. Furthermore, these species also give a strong correlation with protons from the linker (at δ_DQ_ {^1^H} ∼ 10.3 ppm), suggesting that they are somewhat removed from the defect site. The remaining defect proton environments are thus likely to be −OH sites (at δ_SQ_ {^1^H} ∼ 1.5 ppm), and cross-correlations with OH_2_ environments suggest that these sites are all in close proximity and remain protonated upon the attachment of Cu sites. A quantitative comparison (Figure S11 and Table S3) between the defect sites of UiO-66 and Cu/UiO-66 suggests that displacement of adsorbed water and/or dehydroxylation of the {Zr_6_} clusters during Cu attachment and subsequent H_2_-reduction leads to a ∼4-fold increase in the relative amount of protons at the defect sites (δ {^1^H} < 2.5 ppm). The autocorrelation peak of the lowest-shifted ^1^H species of Cu/UiO-66 (at δ_SQ_ {^1^H} ∼ 1.5 ppm) and its cross-correlation to OH_2_ environments indicates that Cu is coordinated at defect sites as {Zr–O(H)–Cu(OH_2_)–O(H)–Zr}. Quantitatively, this coordination mode appears approximately once every two {Zr_6_} clusters. The other coordination modes of Cu sites are {Zr–O–Cu(OH_2_)–O–Zr} and {Zr–O–Cu–O–Zr}. These coordination modes were confirmed by ^1^H hyperfine sublevel correlation (HYSCORE) EPR spectra (Figure S12 and Table S4), which reveal a coordinated H_2_O molecule in the plane perpendicular to the *z* axis of the electronic *g* matrix (the latter being perpendicular to the coordination plane) with a Cu···H distance of ∼2.3 Å.

### Studies of Catalytic Performance

**Figure 3:**
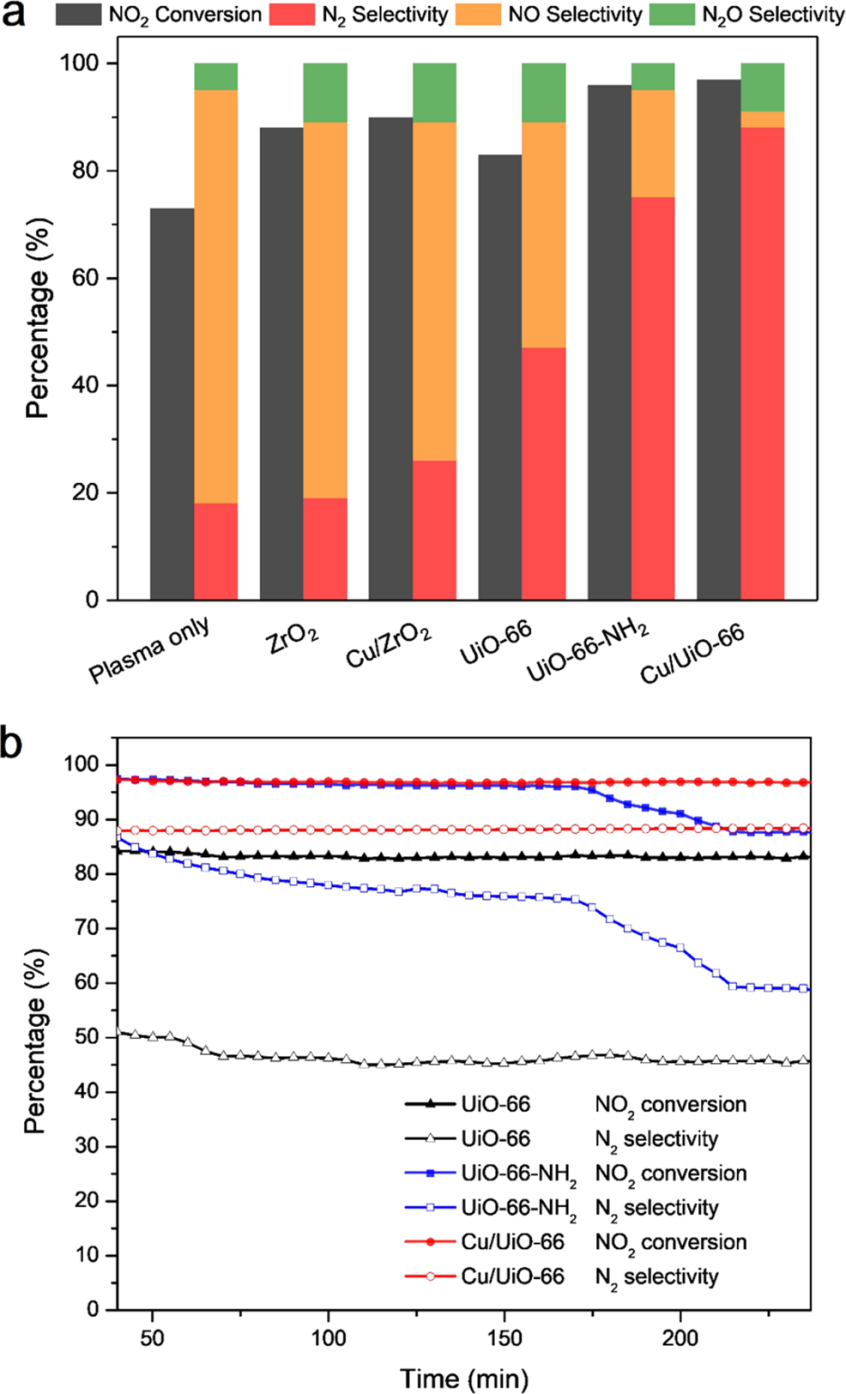
(a) Catalytic performance (NO_2_ conversion and product selectivities) over different catalysts under steady-state NTP conditions. (b) Time-on-stream plots of NO_2_ conversion and N_2_ selectivity over MOF-based catalysts (UiO-66, UiO-66-NH_2_, and Cu/UiO-66) under NTP conditions (0.4 kJ L^–1^, 10 kHz, 500 ppm of NO_2_ diluted in He, 25 °C, and atmospheric pressure).

To investigate the catalytic performance of UiO-66, UiO-66-NH_2_, and Cu/UiO-66 for NTP-assisted decomposition of NO_2_, a simulated gas flow containing 500 ppm of NO_2_ diluted in helium at a total flow rate of 100 mL min^–1^ was used as the feed gas for a proof-of-concept study. Without a catalyst, the gas phase NTP alone exhibited a conversion of NO_2_ (*C*_NO_2__) of 73% with a low selectivity of N_2_ (*S*_N_2__) of 18%, with the majority of NO_2_ being converted to NO, which is another major air pollutant that can be oxidized to NO_2_ in air (Figure 3, Table 1, Entry 1). All three MOF catalysts can improve the *C*_NO_2__ and *S*_N_2__ (Figure 3, Table 1, Entries 2–5). UiO-66 showed a steady *C*_NO_2__ of 83% with a S_N_2__ of 47% over 240 min (Figure 3b).

**Table 1:** Summary of the NO_2_ Conversion and Product Selectivities over Different Catalysts^a^

catalyst	NO_2_ conversion (%)	N_2_ selectivity (%)	NO selectivity (%)	N_2_O selectivity (%)
empty tube	73	18	77	5
UiO-66	83	47	42	11
UiO-66 (H_2_-reduced)	85	50	40	10
UiO-66-NH_2_	96	75	20	5
Cu/UiO-66	97	88	3	9
ZrO_2_	88	19	70	11
Cu/ZrO_2_	90	26	63	11

a Reaction conditions: 0.4 kJ L^–1^, 10 kHz, 500 ppm of NO_2_ diluted in He, 25 °C, and atmospheric pressure.

The NH_2_-functionalized UiO-66-NH_2_ showed improvements to the catalytic activity observed for UiO-66, with the *C*_NO_2__ and *S*_N_2__ reaching 96% and 75%, respectively. However, a notable deactivation of UiO-66-NH_2_ occurred after 170 min, where the *C*_NO_2__ and *S*_N_2__ dropped gradually to 88% and 59%, respectively, after 240 min (Figure 3b). The *operando* DRIFTS spectrum of UiO-66-NH_2_ exposed to NO_2_ under plasma activation (Figure S20) shows a new band at 2280 cm^–1^ assigned to a diazonium salt^28^ and a decrease in intensity of the bands at 3514 and 3398 cm^–1^ assigned to asymmetric and symmetric N–H stretches. These observations suggest that the −NH_2_ group acts as a sacrificial agent in this reaction, with the deactivation of the catalyst being driven by the formation and dissociation of the diazonium ions upon irreversible reaction between NO_2_ and the −NH_2_ groups of UiO-66-NH_2_. Despite the −NH_2_ sites being consumed during the reaction, the overall structure of the framework in UiO-66-NH_2_ was retained, as confirmed by PXRD (Figure S14).

**Figure 4:**
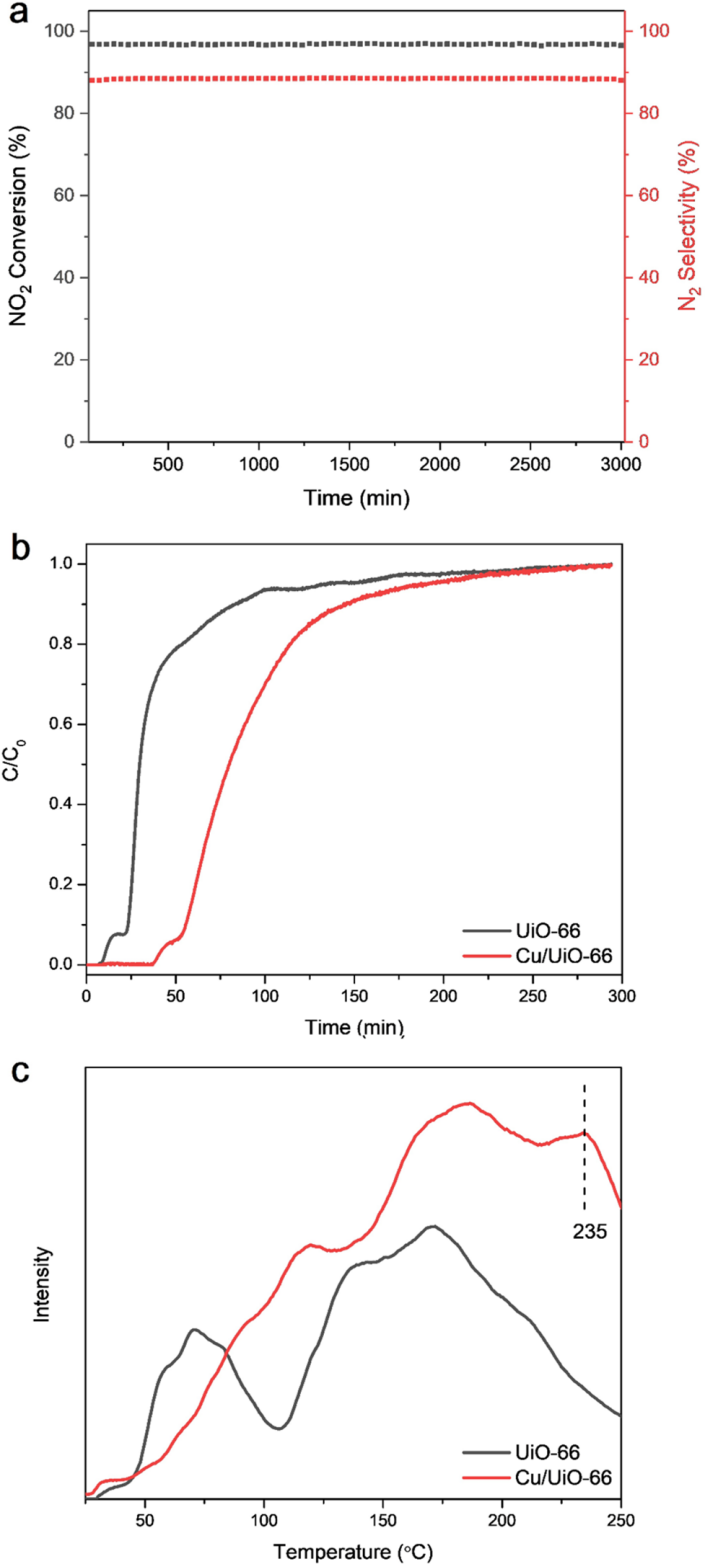
(a) Time-on-stream test of Cu/UiO-66 under NTP conditions. The catalytic efficiency remained constant for 50 h of reaction under continuous flow. (b) Dynamic breakthrough experiments of UiO-66 and Cu/UiO-66 using the same NO_2_/He gas feed at 25 °C. (c) NO_2_-TPD plots of UiO-66 and Cu/UiO-66.

Of the three catalysts, Cu/UiO-66 exhibits the highest catalytic efficiency, with the *C*_NO_2__ and *S*_N_2__ reaching 97% and 88%, respectively (Figure 3 and Table 1, Entry 5). Time-on-stream (ToS) tests further demonstrated the excellent catalytic stability of Cu/UiO-66 with high values for *C*_NO_2__ and *S*_N_2__ retained for over 50 h (Figure 4a). Importantly, Cu/UiO-66 exhibited a superior TOF of 6.1 h^–1^ compared with state-of-the-art Cu-based SCR catalysts, including those using NH_3_ as reductant and operating at elevated temperatures (Table 2). For example, a Cu-exchanged SSZ-13 zeolite^29^ shows a TOF value of 1.9–2.2 h^–1^ at 250–550 °C and Cu-MOF-74^30^ demonstrates a TOF of 0.28 h^–1^ at 230 °C. Significantly, the excellent catalytic activity of Cu/UiO-66 was achieved without the use of NH_3_ or heating. In contrast, Cu/ZrO_2_, prepared through a similar method as for Cu/UiO-66 (Section S1.1), showed a much lower catalytic efficiency, with values of 90% and 26% for *C*_NO_2__ and *S*_N_2__, respectively( Figure 3a and Table 1, Entry 7), indicating that the nature of atomically dispersed Cu sites within Cu/UiO-66 plays a crucial role in its observed activity. Although NTP-activated deNO_*x*_ systems have been reported,^31−33^ they generally suffer from poor catalytic efficiency, low TOF, and/or stability.

**Table 2:** Summary of Values of Turnover Frequency (TOF) Reported for Cu-Based Catalysts for NO_*x*_ Conversion under NTP Conditions and Thermal Conditions with Reducing Agents^a^

catalyst	temperature (°C)	GHSV (10^3^h^–1^)	NO_*x*_ concentration (ppm)	NO_*x*_ conversion (%)	TOF (h^–1^)	ToS (h)
Cu/UiO-66@NTP [this work]	25	150	500	97.0	6.1	50
HKUST-1@NTP^31^	25	75	500	97.9	0.27	0.50
Cu/ZSM-5@NTP-NH_3_^32^	25	60	500	21.0	0.62	
Cu/ZSM-5@NTP-NH_3_^32^	180	60	500	60.0	1.8	
Cu/MFM-300(Al)@NTP^33^	25	150	500	96	2.9	3.8
Cu-SSZ-13@thermal-NH_3_^29^	250	30	350	95.0	2.2	
Cu-SSZ-13@thermal-NH_3_^29^	550	30	350	83.0	1.9	
Cu-MOF-74@thermal-NH_3_^30^	230	50	1000	97.8	0.28	24
Cu/SAPO-34@thermal-NH_3_^46^	210	36	500	90.0	1.0	
Cu/SAPO-34@thermal-NH_3_^46^	360	36	500	100	1.1	
Cu-ZSM-5@thermal-NH_3_^47^	150	100	500	48.0	2.4	
Cu-ZSM-5@thermal-NH_3_^47^	300	100	500	100	4.9	
HKUST-1@thermal-NH_3_^48^	280	50	1000	95.5	0.26	
CuO@thermal-NH_3_^49^	270	50	1000	97.2	0.12	25

a Note: GHSV = gas hourly space velocity. ToS = time-on-stream. TOF = turnover frequency.

To explore possible sample degradation, the used Cu/UiO-66 catalyst was characterized by EPR, XPS, PXRD, DRIFTS, and N_2_ adsorption isotherms. The X-band EPR spectrum of the used catalyst confirms (Figure S16) the retention of atomically dispersed Cu sites, with no evidence of aggregated species. The EPR spectrum also shows a small increase in the intensity of Cu(II) signal after reaction (Figure S8). XPS spectra indicate that the ratio of Cu(II)/Cu(I) increases after reaction, but with the majority of Cu sites still remaining as Cu(I) in the used catalyst (Figure S17). Little difference was observed in the PXRD patterns or DRIFTS spectra of fresh and used catalysts (Figures S15 and S18). N_2_ adsorption isotherms at 77 K indicate a small reduction (∼10%) in the surface area of Cu/UiO-66 after reaction (Figure S19).

### Studies of the Reaction Mechanism

The adsorption of NO_2_ onto the catalyst is widely considered as the rate-determining step for the reduction of NO_2_,^34^ and dynamic breakthrough experiments were therefore carried out with UiO-66 and Cu/UiO-66 at 25 °C (Figure 4b). Notably, the dynamic adsorption capacity of NO_2_ in UiO-66 increases from 2.02 to 3.96 mmol g^–1^ on incorporation of Cu sites, and the temperature-programmed desorption (TPD) profiles of NO_2_-adsorbed onto UiO-66 and Cu/UiO-66 show the presence of stronger binding sites for NO_2_ in the latter with an additional desorption peak at 235 °C (Figure 4c). Thus, both breakthrough and TPD experiments confirm the stronger adsorption of NO_2_ in Cu/UiO-66 than in UiO-66, presumably contributing to the observed superior catalytic performance.

**Figure 5:**
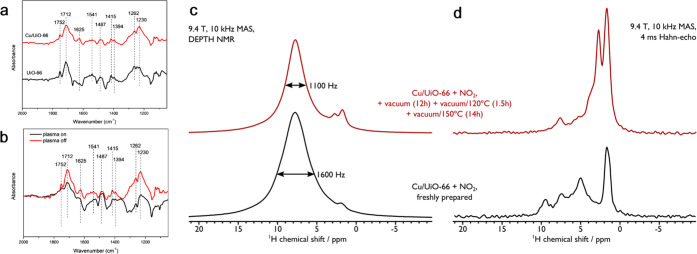
(a) *In situ* DRIFTS spectra of adsorbed NO_2_ on Cu/UiO-66 (red) and UiO-66 (black). (b) *Operando* DRIFTS spectra over Cu/UiO-66 with plasma on (black) and off (red); 500 ppm of NO_2_ in He was used as a feed gas. All the DRIFTS spectra were recorded at a resolution of 4 cm^–1^; the spectra of bare MOF samples were subtracted as the background. (c) ^1^H DEPTH and (d) ^1^H-Hahn-echo MAS NMR spectra of Cu/UiO-66 dosed with NO_2_ for 15 min (black) and the same sample after NO_2_ desorption (red). A further desorption study can be found in the Supporting Information.

*In situ* DRIFTS spectroscopy confirmed the adsorption of NO_2_, resulting in several new bands at ∼1541, 1487, 1415, 1394, 1262, and 1230 cm^–1^ (Figure 5a) assigned to various monodentate, bidentate, and bridging nitro/nitrate species^35−37^ adsorbed on catalyst surfaces with partially overlapping vibrational bands. The band at 1712 cm^–1^ is associated with adsorbed NO on the −OH defect sites in both UiO-66 and Cu/UiO-66, and the notable broadening and increase of intensity of this band in Cu/UiO-66 is due to the additional contribution from the formation of {Cu(I)···NO} species.^38,39^ The adsorption of NO onto the −OH defect sites was further confirmed by the depletion of the O–H stretching vibration band at 3675 cm^–1^ (Figure S21). Interestingly, an additional band was observed at 1625 cm^–1^ for NO_2_-loaded Cu/UiO-66, which could originate from adsorbed NO_2_ or NO species on Cu sites.^35,40,41^ Thus, the formation of {Cu···NO_2_} adducts on Cu/UiO-66 greatly accelerates the adsorption and reduction of NO_2_. The EPR spectroscopic studies of Cu/UiO-66 and NO_2_-loaded Cu/UiO-66 also show differences in both the *g*- and Cu hyperfine *A*-tensors (Figure S22), consistent with binding of NO_*x*_ at Cu sites. *Operando* DRIFTS experiments were carried out as a function of plasma on–off and reaction time (Figure 5b). Apparent reductions of FTIR bands of various adsorbed nitro/nitrate species, {Cu(I)···NO}, and {Cu···NO_2_} adducts were observed upon the ignition of plasma, indicating the rapid conversion of these intermediates over Cu/UiO-66. It is worth noting that, in the present system, the activation effects of NTP coupled to the adsorption of NO_2_ by the catalyst appear to work synergistically.

*In situ*^1^H MAS NMR spectra were recorded for Cu/UiO-66 upon the adsorption of NO_2_ to investigate the adsorbates as well as the local structural changes of the catalyst. The adsorption of NO_2_ has substantial effects (cf. Figures 2a and 5c) with the broadening of the ^1^H NMR signals from the ligand (at δ {^1^H} ∼ 8 ppm) and a decrease in the intensity of signals due to the defect sites (at δ {^1^H} < 2.5 ppm). To investigate the broadening, a ^1^H Hahn-echo experiment^42^ was employed with a relatively long total echo duration (Figure 5d) as this will remove homogeneously broadened signals [e.g., from ^1^H centers near Cu(II) sites or with strong dipolar couplings]. Subsequently, the adsorbed NO_2_ was removed using vacuum/heating treatment (Figure 5c and Figure S23), which resulted in reduced line width of the ^1^H resonance for the ligand and an increased intensity for the −OH species. Both NMR spectroscopic experiments indicate that Cu(II) is not responsible for the line broadening observed upon the adsorption of NO_2_ but is a result of increased structural heterogeneity that facilitates the adsorption of NO_2_ onto the MOF interior and, in addition, NO_2_ binds to −OH and Cu sites, as revealed by the DRIFTS experiments. Interestingly, the increased resolution in the ^1^H Hahn-echo MAS NMR spectrum of NO_2_-adsorbed Cu/UiO-66 (Figure 5d, bottom) reveals a high-shifted ^1^H signal at δ {^1^H} ∼ 9.6 ppm, along with adsorbed bulk water at δ {^1^H} ∼ 5.3 ppm; it should be noted that this experiment is not quantitative. Upon the removal of NO_2_, the high-shifted ^1^H signal and that of bulk water mostly disappear, while low-shifted −OH/OH_2_ signals emerge (Figure 5d, top). The high-shifted ^1^H signals (at δ {^1^H} ∼ 9.6 ppm) can be ascribed to nitric/nitrous acid species.^43,44^ The 2D ^1^H homonuclear dipolar correlation spectrum of Cu/UiO-66 with NO_2_ (Figure S24) displays correlations between these acidic protons and protons of the ligand but not with water or defect protons. Notably, this indicates that an intermediate in the reduction of NO_2_ by Cu/UiO-66 could be nitric/nitrous acid and that it adsorbs, most likely through hydrogen-bonding, to the ligand. This is corroborated with data from ^13^C MAS NMR (Figure S25), which shows a shift in the carboxylate ^13^C signal upon adsorption of NO_2_.^45^

## Conclusions

The development and application of new efficient technologies to enable the precise identification of single metal sites and their roles in catalysis are important targets. We have undertaken a comprehensive study of the local structure of the single-atom Cu sites in Cu/UiO-66, thus enabling elucidation of their critical role in the reduction of NO_2_. The combination of the single-atom catalyst Cu/UiO-66 and NTP leads to the direct decomposition of NO_2_ to N_2_ at room temperature. Cu/UiO-66 exhibits unprecedented catalytic efficiency and excellent stability. Atomically dispersed Cu sites anchored at the defect sites of the UiO-66 scaffold prevents the Cu atoms from migrating and sintering during reaction and are responsible for the superior catalytic performance. In Cu/UiO-66, the adsorption of NO_2_ occurs at the Cu and hydroxyl sites, forming nitro/nitrate and related intermediates, which readily transform on activation with NTP to regenerate accessible active sites on the catalyst surface. The excellent catalytic performance of Cu/UiO-66 originates from the synergistic effects between atomically dispersed Cu sites and the framework porosity, providing multiple binding sites to promote the adsorption and conversion of NO_2_. New controllable deNO_*x*_ processes integrating single-atom catalysts with a NTP technique could provide an efficient solution to the mitigation of NO_*x*_ emissions.

## Supporting Information Available

Additional experimental details, PXRD and NPD data, N_2_ adsorption isotherms, TGA plots, SEM and HAADF-STEM images, EPR, UV−Vis, solid-state NMR, XPS, Auger and DRIFTS spectra, Figures S1−S25 and Tables S1−S6 (PDF)

## Accession Codes

CCDC 2054313 and 2054314 contain the supplementary crystallographic data for this paper.
