# Supplementary material for: Atomically Dispersed Copper Sites in a Metal–Organic Framework for Reduction of Nitrogen Dioxide
Source: J Am Chem Soc. 2021 Jul 19;143(29):10977–85. doi: 10.1021/jacs.1c03036 (PMC8323097; doi:10.1021/jacs.1c03036)
Supplement: Supplementary file 1 — ja1c03036_si_001.pdf [file ja1c03036_si_001.pdf]

Supporting Information

Atomically dispersed copper sites in a metal-organic framework for reduction of nitrogen dioxide

Yujie Ma,[1] Xue Han,[1] Shaojun Xu,[1,2,8] Zi Wang,[1] Weiyao Li,[1] Ivan da Silva,[3] Sarayute Chansai,[4] Daniel Lee,[4]* Yichao Zou,[5] Marek Nikiel,[5] Pascal Manuel,[3] Alena M. Sheveleva,[1,6] Floriana Tuna,[1,6] Eric J. L. McInnes,[1,6] Yongqiang Cheng,[7] Svemir Rudić,[3] Anibal J. Ramirez-Cuesta,[7] Sarah J. Haigh,[5] Christopher Hardacre,[4] Martin Schröder[1]* and Sihai Yang[1]*

1. Department of Chemistry, University of Manchester, Manchester M13 9PL, United Kingdom

2. UK Catalysis Hub, Research Complex at Harwell, Rutherford Appleton Laboratory, Harwell OX11 0FA, United Kingdom

3. ISIS Facility, STFC Rutherford Appleton Laboratory, Chilton, Oxfordshire OX11 0QX, United Kingdom

4. Department of Chemical Engineering and Analytical Science, University of Manchester, Manchester M13 9PL, United Kingdom

5. Department of Materials, University of Manchester, Manchester M13 9PL, United Kingdom

6. Photon Science Institute, University of Manchester, Oxford Road, Manchester M13 9PL, United Kingdom

7. Neutron Scattering Division, Neutron Sciences Directorate, Oak Ridge National Laboratory, Oak Ridge, Tennessee 37831, United States

8. School of Chemistry, Cardiff University, Main Building, Park Place, Cardiff CF10 3AT, United Kingdom

List of Contents

1. Experimental section

   1.1 Synthesis of catalysts

   1.2 Characterisation of catalysts

2. Experimental setup for catalytic testing

3. Characterisation of used catalysts

4. Studies of the reaction mechanism

   4.1 *In situ* and *operando* DRIFTS spectra

   4.2 *In situ* EPR spectroscopy

   4.3 *In situ* MAS NMR spectroscopy

5. References

1. Experimental section

1.1 Synthesis of catalysts

Synthesis of UiO-66. All the reagents were used as received from commercial suppliers without further purification. UiO-66 was synthesised through solvothermal method according to the literature[1]. Typically, 125 mg of terephthalic acid and 167 mg of zirconium tetrachloride were dissolved in a mixture solution containing 50 mL of N,N-dimethylformamide (DMF) and 3.5 mL of formic acid. The mixture was transferred into a teflon lined bomb, sealed and heated at 120 °C for 24 h. The white product was collected by filtration and washed with DMF 4 times over 24 h (20 mL × 4), and then 6 times with acetone over a 48 h period (20 mL × 6). UiO-66 was dried under dynamic vacuum before further use.

Synthesis of UiO-66-NH$_2$. The method to synthesise UiO-66-NH$_2$ was the same as that for the synthesis of UiO-66, except 2-aminoterephthalic acid was used in place of terephthalic acid.

Synthesis of Cu/UiO-66. Cu/UiO-66 was prepared according to a reported method[1]. Typically, MOF powder (about 500 mg of UiO-66) was added to a solution containing $CuCl_2$ (500 mg) dissolved in DMF (10 mL). The mixture was sonicated for one minute and then heated at 85 °C for 24 h. The products were collected by centrifugation (10,000 rpm, 3 min), washed 4 times with DMF during 24 h (30 mL × 4), and 6 times with acetone over a 48 h period (30 mL × 6). Finally, Cu/UiO-66 was dried with dynamic vacuum before further use. Before NTP-assisted deNO$_x$ reaction, Cu/UiO-66 was reduced in a tube furnace with 5% H_2 flow (diluted in Ar) at 250 °C for 2 h. The sample of H_2-reduced Cu/UiO-66 was stored under Ar to prevent slow oxidation in air, and packed into a fixed-bed reactor in a glovebox for catalysis experiments.

Synthesis of Cu/ZrO$_2$. Cu/ZrO$_2$ was prepared using the same method for the preparation of Cu/UiO-66, except that ZrO_2 was used in place of UiO-66.

1.2 Characterisation of catalysts

Powder X-ray diffraction (PXRD) patterns were recorded using a Philips X'pert X-ray diffractometer (40kV and 30 mA) using Cu Kα radiation (λ = 1.5406 Å). N_2 adsorption isotherms were measured on a Micromeritics 3-Flex instrument at 77 K. The samples were activated under dynamic vacuum at 423 K for 16 h before measuring. The crystal morphology was characterized by scanning electron microscopy (SEM) on a HITACHI SU8100. High-angle annular dark-field scanning transmission electron microscopy (HAADF-STEM) imaging and energy dispersive X-ray spectroscopy (EDX) mapping was performed using a Titan ChemiSTEM transmission electron microscope operated at 200 kV with a probe current of 40-360 pA and a convergence angle of 21 mrad. EDX elemental mapping was acquired by scanning for 5-20 minutes (30us dwell time) and

quantified using a standard-less Cliff Lorimer approach in the Bruker Esprit software. Elemental analysis for the catalysts was measured with a Thermo iCap 6300 inductively coupled plasma optical emission spectroscopy (ICP-OES), and TGA was measured under air at a flow rate of 10 $mL \cdot min^{-1}$. UV-Vis spectra were recorded on a UV-Vis spectrophotometer (Shimadzu, UV 2600). XPS spectra were measured using a Kratos Axis Ultra instrument equipped with a monochromatic Al ka X-ray source (E = 1486.6 eV). A charge neutraliser was used to minimise charging and spectra are aligned on the binding energy scale relative to the hydrocarbon C-C/C-H peak at 284.8 eV. Spectra were fitted using the CASA XPS software using Voigt-like peak shapes. Spin-orbit splitting ratios and splitting energies are constrained to obtain physically meaningful fits.

Neutron powder diffraction (NPD) experiments were carried out at WISH, a long wavelength powder and single crystal neutron diffractometer at the ISIS Facility at the Rutherford Appleton Laboratory, UK[2]. Prior to NPD experiments, the sample was activated by heating at 393 K under dynamic vacuum for 16 h, and the desolvated sample was then transferred into a cylindrical vanadium sample cell with an indium seal. The sample was further degassed at 373 K under dynamic vacuum to remove traces of adsorbed moisture. The temperature during data collection was controlled using a He cryostat (7 ± 0.2 K). The quality of the Rietveld refinements has been assured with low goodness-of-fit (gof) factors, low weighted profile factors (Rwp) and well-fitted patterns with reasonable isotropic displacement factors (Beq). Inelastic neutron scattering (INS) experiments were performed at TOSCA neutron spectrometer at the ISIS Facility[3].

CW EPR spectra of Cu/UiO-66 were measured with a Bruker EMX 300 EPR spectrometer equipped with a high sensitivity X-band (ca. 9.4 GHz) resonator and a liquid He cryostat. Field corrections were applied by measuring relevant EPR standards (Bruker Strong Pitch and DPPH). For accuracy, the tube size and tube position in the cavity were kept constant. Parallel mode EPR data were collected with a dual-mode EPR resonator ER 4116DM at the microwave frequency of ca. 9.4 GHz (perpendicular mode EPR is measured at the frequency of ~9.6 GHz). To obtain the EPR sample, Cu/UiO-66 (25 mg) after H_2 reduction was quickly transferred into a J. Young X-band EPR tube (4 mm). The sample was purged by argon gas flow for 2 h and evacuated at 10^{-5} mbar for 2 h at room temperature. For the NO_2 adsorption studies, the same sample in a J. Young X-band EPR tube was dosed with NO_2 gas at 1 bar. For comparison of the intensities of Cu(II) signals before H_2 reduction, after H_2 reduction, and after plasma reaction, the same amount of Cu/UiO-66 (before H_2 reduction) were used to prepare the EPR samples. The spectra were normalised by mass, with identical sample positioning and instrumental settings. CW EPR spectra were recorded at microwave power of 0.7-2.2 mW,

modulation frequency 100 kHz, and modulation amplitude 5 G. Pulsed EPR measurements were performed at X-band (ca. 9.7 GHz) on a Bruker ElexSys E580 spectrometer. The microwave frequency was measured with a built-in digital counter and the magnetic field was calibrated using a Bruker strong pitch reference sample. EPR echo-detected (ED) spectra were measured at X-band using a Hahn echo sequence, $\pi/2 - \tau - \pi - \tau$ – echo, with $\pi/2$ and π pulse lengths of 16 and 32 ns, respectively, and an inter-pulse delay τ of 150 ns. HYSCORE[4] spectra were measured at X-band using standard pulse sequence ($\pi/2 - \tau - \pi/2 - T1 - \pi - T2 - \pi/2 - \tau$ – echo); the length of mw pulses was $\pi/2 = 16$ ns and $\pi = 26$ ns. The time delay between first two pulses was taken as $\tau = 140$ ns and $\tau = 200$ ns, and starting values of T1 and T2 incrementing times were 100 ns. The (128x128) HYSCORE data array was recorded with a time increment of 16 ns, and then two-dimensional Fourier transform (FT) magnitude spectra were calculated. Simulation of the EPR spectra was performed with the EasySpin/MATLAB toolbox, which employs the exact diagonalization of the spin Hamiltonian matrix[5]. The difference between the two Cu isotopes (^{63}Cu and ^{65}Cu) is included in the simulation program but the effect is not resolved at the linewidths observed.

MAS NMR spectra were recorded using two regimes. Moderate-field experiments employed a Bruker 9.4 T (400 MHz ^{1}H Larmor frequency) AVANCE III spectrometer equipped with a 4 mm HFX MAS probe. Experiments were acquired at ambient temperature using a MAS frequency of 10 kHz. ^{1}H-pulses of 100 kHz were used and ^{13}C spin-locking at ~25 kHz was applied for 2 ms, with corresponding ramped (70-100 %) ^{1}H spin-locking at ~50 kHz for CP experiments with 100 kHz of SPINAL-64[6] heteronuclear ^{1}H decoupling used throughout signal acquisition. Samples were treated and packed into 4 mm o.d. zirconia rotors under inert conditions and sealed with a Kel-F rotor cap fitted with an O-ring. For NO_2 adsorption/desorption, the rotor packed with Cu/UiO-66 was opened under inert conditions and the sample remained in this container during the applied treatments, before subsequent resealing with the O-ring Kel-F cap. High-field NMR spectra, recorded at the UK High-Field Solid-State NMR Facility, employed a Bruker 20.0 T (850 MHz ^{1}H Larmor frequency) AVANCE NEO spectrometer equipped with a 1.3 mm HXY MAS probe, and was used in double-resonance mode. Experiments were acquired at ambient temperature using a MAS frequency of 60 kHz. ^{1}H-pulses of 100 kHz were used, except during the S_3 recoupling sequence[7,8] where 30 kHz-pulses were required. One full loop of S_3 recoupling was used to reintroduce the homonuclear dipolar interaction between ^{1}H-spins during both excitation and reconversion periods, giving a total mixing time of 267 μs. For the 2D DQ-SQ experiments, 32 transients were acquired for each of 32 complex (STATES-TPPI) rotor-synchronised t_1

increments (2 rotor periods). Samples were treated and packed into 1.3 mm o.d. zirconia rotors under inert conditions and sealed with a Vespel rotor cap.

Molecular dynamics (MD) simulations were performed using CP2K (http://www.cp2k.org)[9], based on the mixed Gaussian and plane-wave scheme[10] and the Quickstep module[11]. The calculation used molecularly optimized Double-Zeta-Valence plus Polarization (DZVP) basis set[12], Goedecker-Teter-Hutter pseudopotentials[13], and the Perdew-Burke-Ernzerhof (PBE) exchange correlation functional[14]. The plane-wave energy cutoff was 400 Ry, and a DFT-D3 level correction for dispersion interactions, as implemented by Grimme *et al*[15], was applied with a cutoff distance of 15 Å. The calculation was performed on Gamma point only, with no symmetry constraint. Structural optimization was performed using the Broyden-Fletcher-Goldfarb-Shannon (BFGS) optimizer, until the maximum force fell below 0.00045 Ry/Bohr (0.011 eV/Å). Finite displacement method was used for the phonon calculation, with incremental displacement of 0.01 Bohr (0.0053 Å). INS spectra were simulated using the OClimax software[16].

In order to measure the NO_2 adsorption ability of the catalysts, dynamic breakthrough experiments were performed at 25 °C. NO_2-TPD experiments were carried out to determine the adsorbate-adsorbent strength of intereaction. Typically, 50 mg of sample was packed in a fixed-bed reactor which was equipped with a Bruker Matrix MG5 FTIR spectrometer (resolution = 0.5 cm^{-1}) as the detector. The sample was then pre-treated under helium flow (100 mL min^{-1}) at 250 °C for 2 h. After activation, the NO_2 breakthrough experiment was carried out at 25 °C. When the outlet concentration of NO_2 was equal to that of inlet, 100 mL min^{-1} of pure helium was used to flush the sample for about 2 h, removing the physically adsorbed NO_2. The NO_2-TPD experiments were then carried out at a heating rate of 5 °C min^{-1} from 25 °C to 250 °C.

Powder X-Ray diffraction (PXRD) patterns of the catalysts

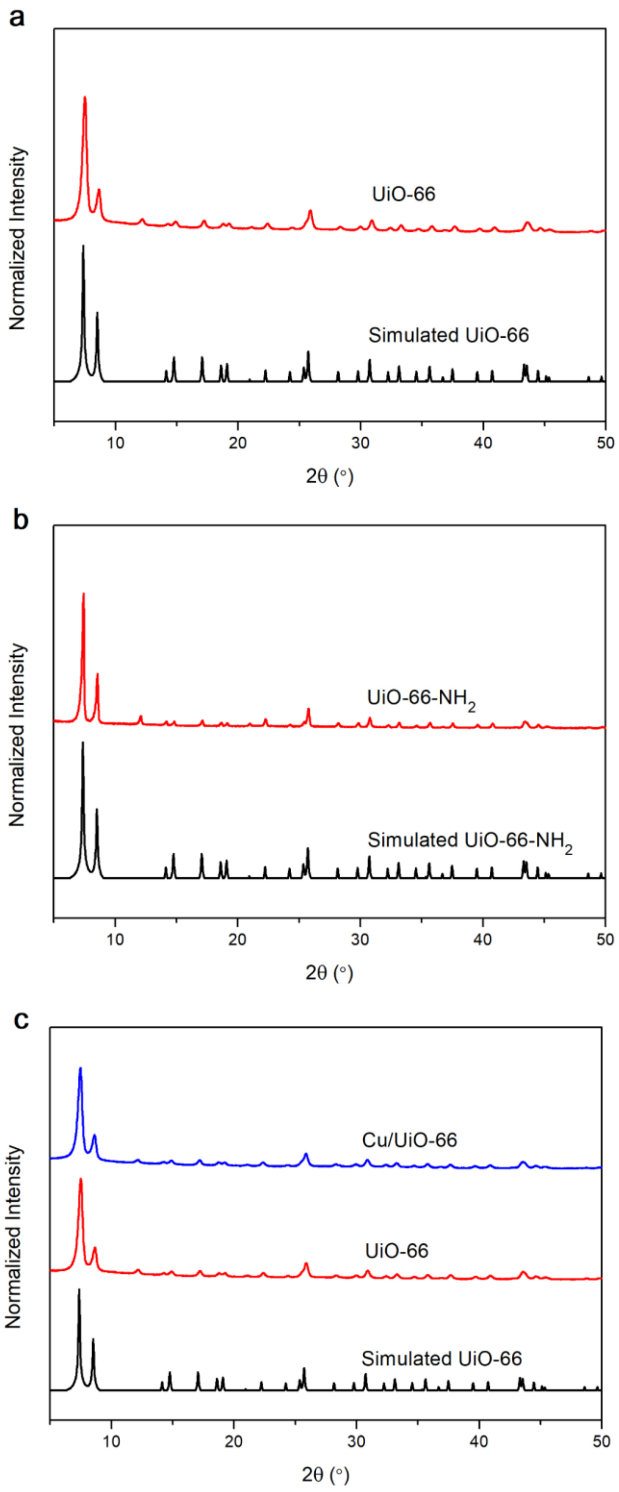

Figure S1. PXRD patterns of (a) UiO-66, (b) UiO-66-NH_2 and (c) Cu/UiO-66.

Nitrogen adsorption isotherms of catalysts

N_2 adsorption isotherms were recorded at 77 K. The samples were activated under dynamic vacuum at 423 K for 16 h.

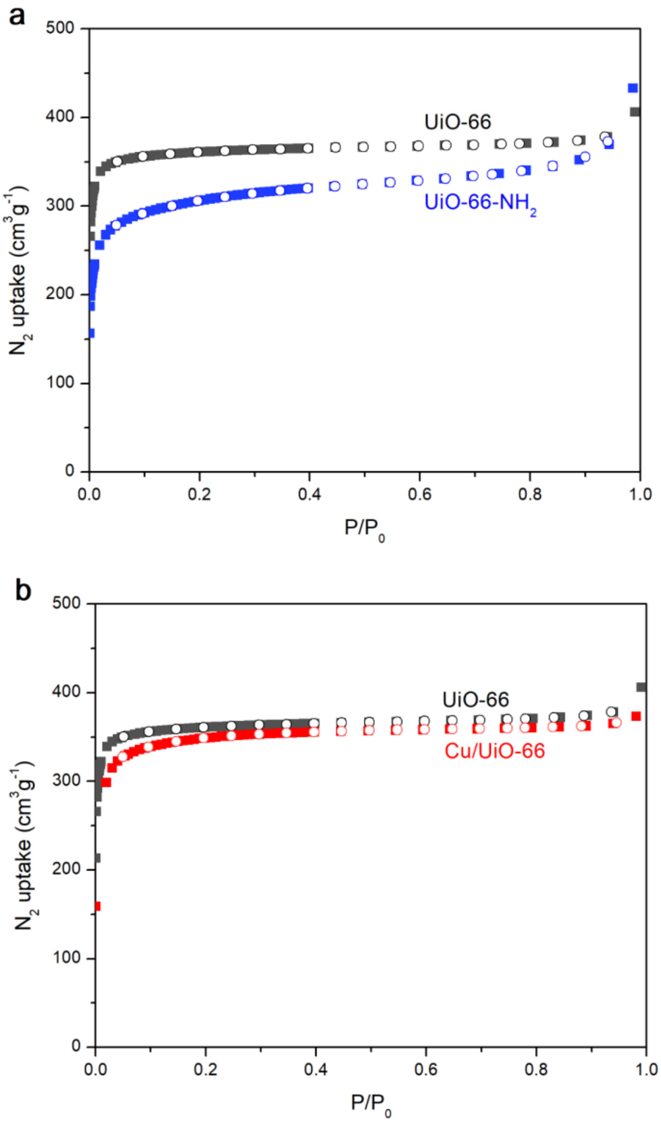

Figure S2. Nitrogen adsorption isotherms of UiO-66, UiO-66-NH_2 and Cu/UiO-66.

Table S1. BET surface areas of UiO-66, UiO-66-NH_2 and Cu/UiO-66.

Catalyst	Calculated BET surface area (m^2 g^{-1})
UiO-66	1084
UiO-66-NH_2	948
Cu/UiO-66	1068

Scanning electron microscopy (SEM) images of catalysts

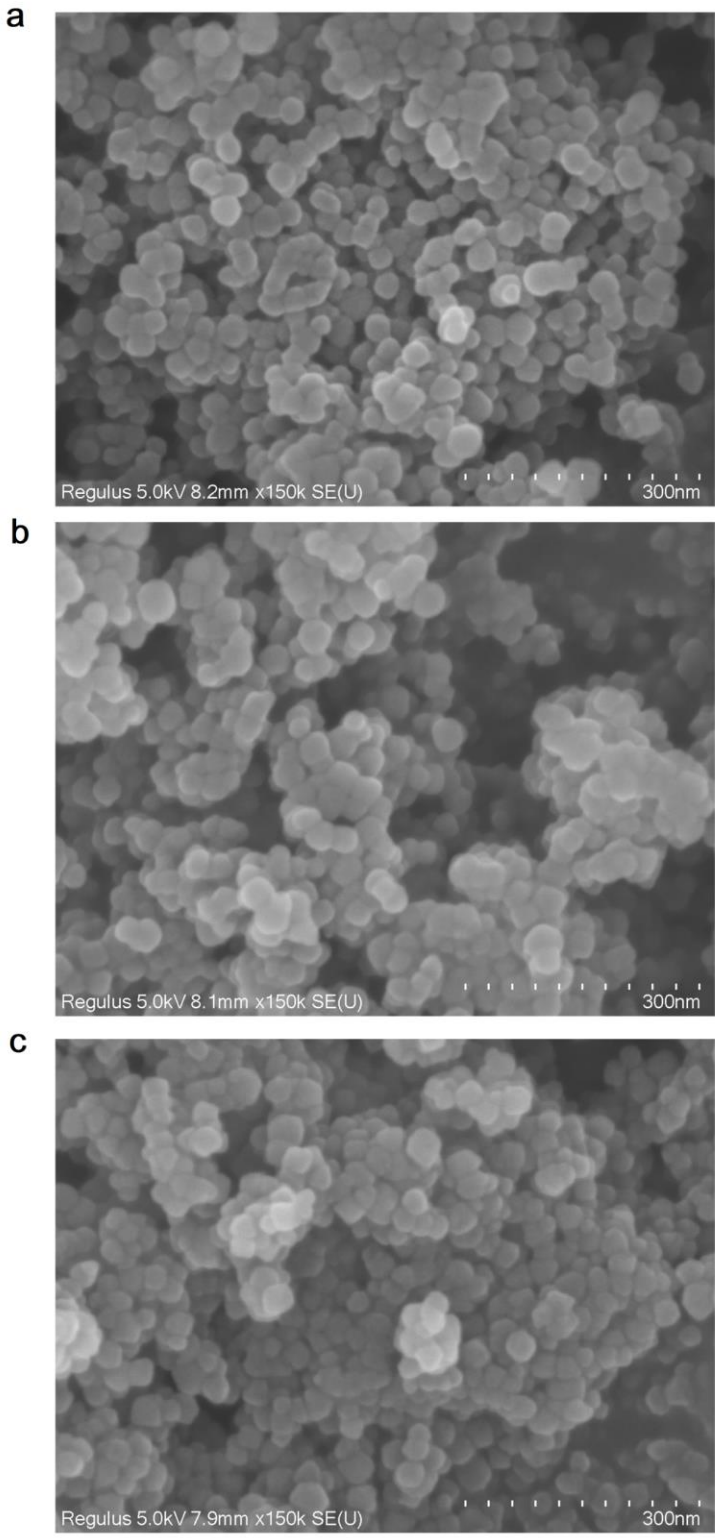

Figure S3. SEM images of (a) UiO-66, (b) UiO-66-NH_2 and (c) Cu/UiO-66.

Thermal gravimetric analysis (TGA) of catalysts

TGA curves were measured with a heating rate of 5 °C min^{-1} under air flow. The samples were dried with dynamic vacuum overnight before analysis.

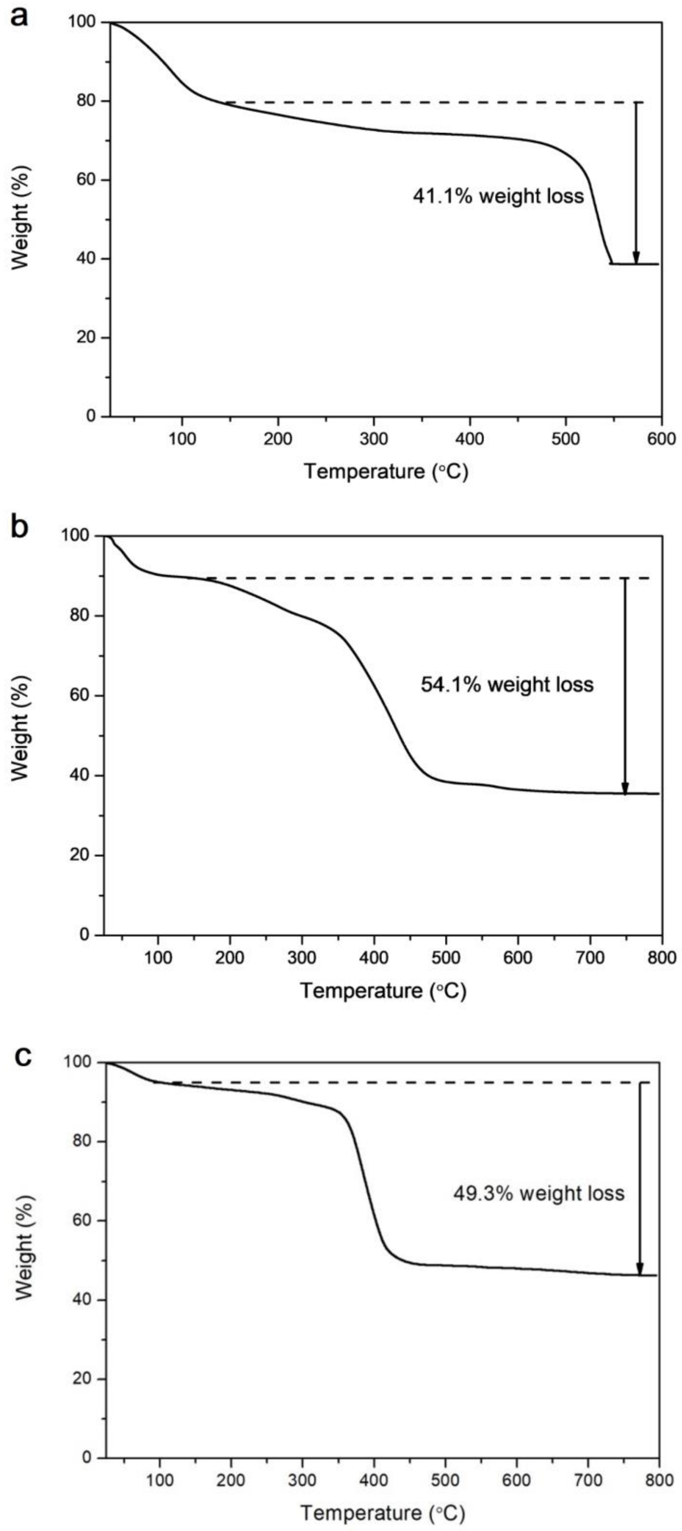

Figure S4. TGA plots of (a) UiO-66, (b) UiO-66-NH_2 and (c) Cu/UiO-66.

Neutron powder diffraction (NPD) patterns of UiO-66 and Cu/UiO-66

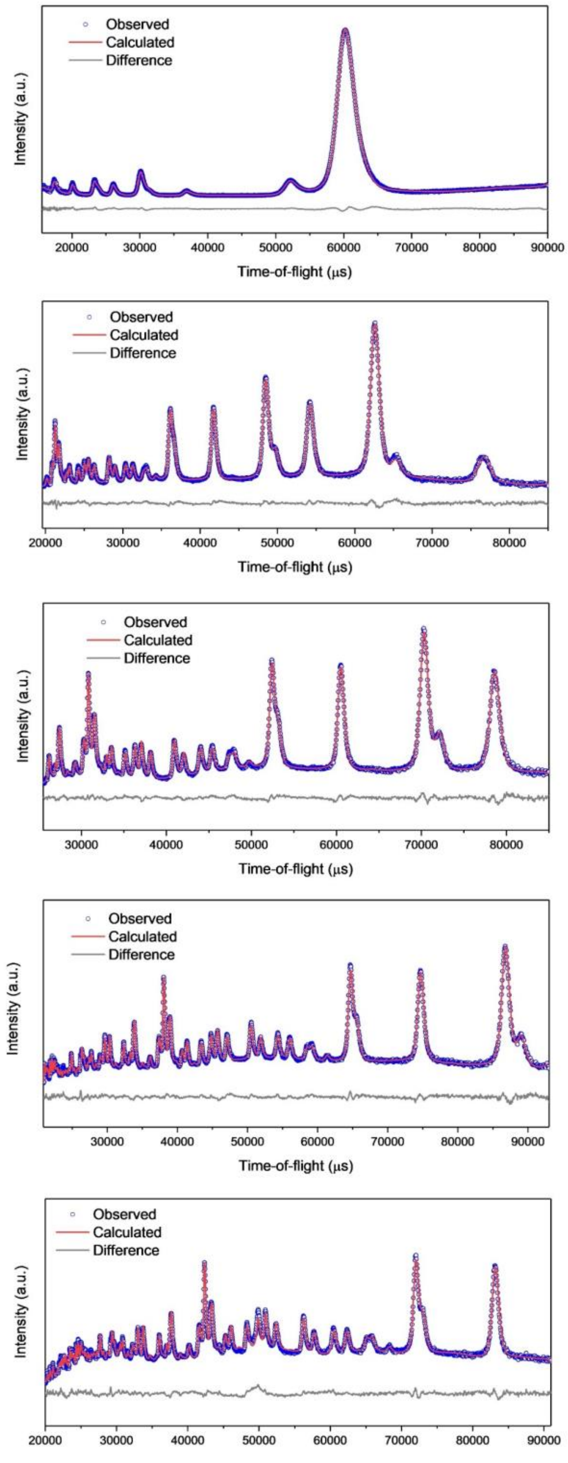

Figure S5. Neutron powder diffraction patterns and Rietveld refinement for UiO-66 (banks 1 to 5). Fitting agreement parameters: R_{exp}=0.35%; R_{wp}=0.82%; R_p=0.68%; Gof=2.31.

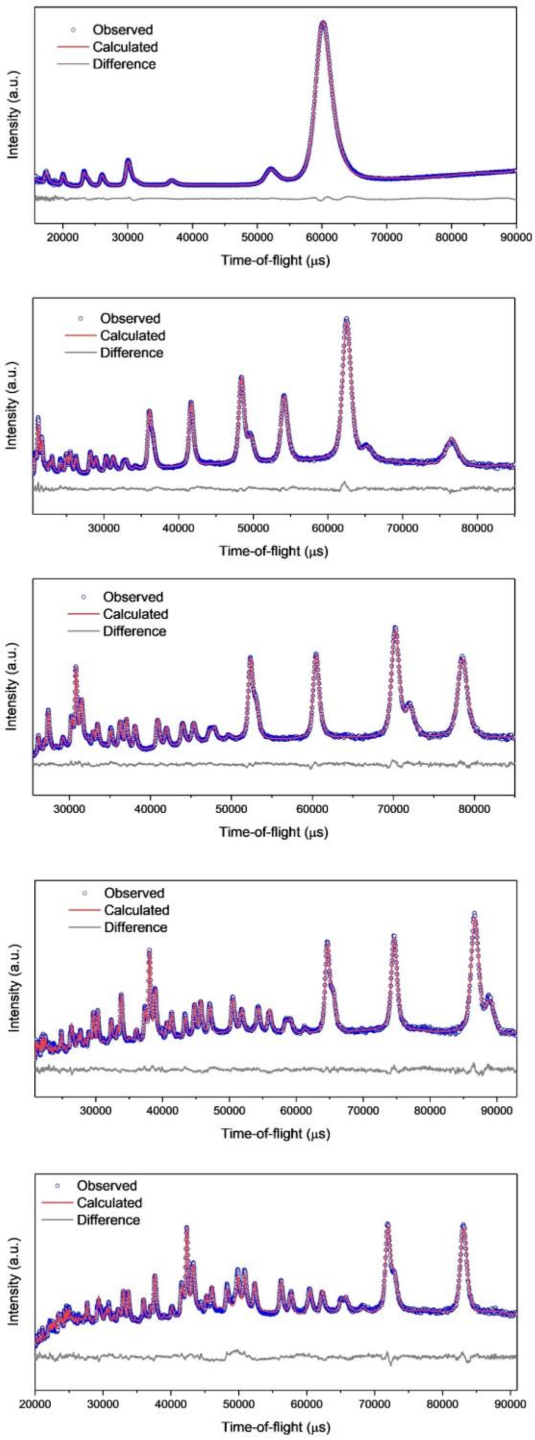

Figure S6. Neutron powder diffraction patterns and Rietveld refinement for Cu/UiO-66 (banks 1 to 5). Fitting agreement parameters: R_{exp}=0.37%; R_{wp}=0.83%; R_p=0.71%; Gof=2.26.

Table S2. Bond distances of UiO-66 and Cu/UiO-66.

Bond distances (Å)	UiO-66	Cu/UiO-66
Zr−O_{2A} (O_{2A} for μ_3−O)	2.068(4)	2.056(4)
Zr−O_{2B} (O_{2B} for μ_3−OH)	2.302(4)	2.242(4)
Zr···Zr	3.515(3), 4.971(1)	3.502(3), 4.952(1)
Zr−O_{1A} (O_{1A} for ligand)	2.288(4)	2.231(2)
C_1−C_2 (C_1 for COO^- and C_2 for benzene ring)	1.510(3)	1.591(3)
C_2−C_3 (both C for benzene ring)	1.436(3)	1.403(3)
C_3−C_3 (both C for benzene ring)	1.451(3)	1.426(3)
Cu−O_{1B}	--	1.88(3)

Electron paramagnetic resonance (EPR) spectra of Cu/UiO-66

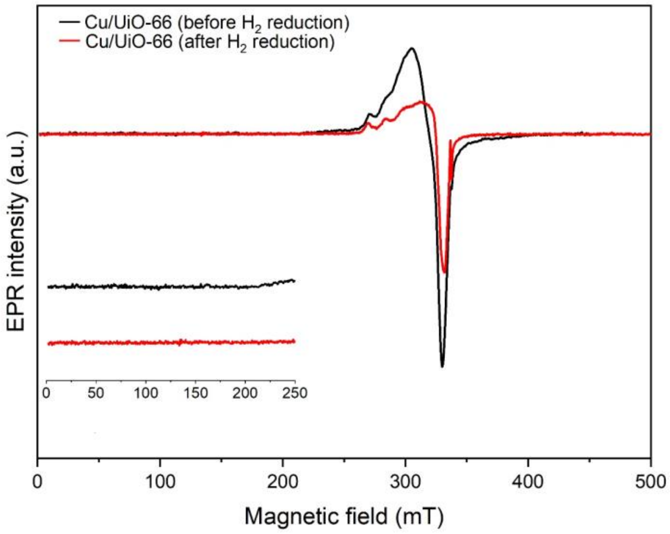

Figure S7. X-band (9.4 GHz) EPR spectra of Cu/UiO-66 before H_2 reduction (black) and Cu/UiO-66 after H_2 reduction (red) at room temperature. Inset: expansion of low-field region, illustrating absence of characteristic signals for binuclear and/or aggregated Cu species ($S \geq 1$).

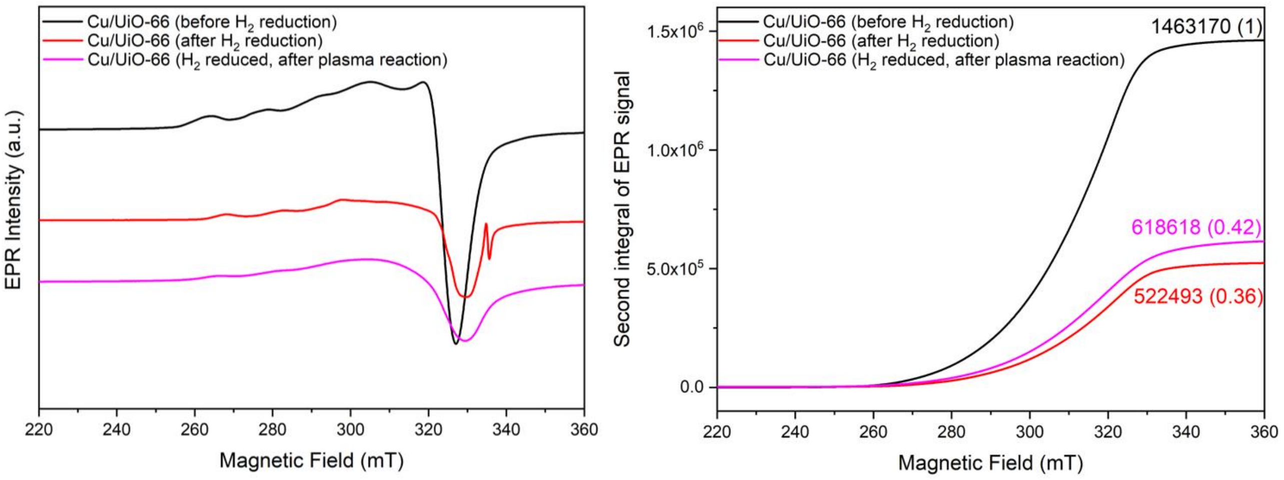

Figure S8. EPR spectra of fresh Cu/UiO-66 (before and after H_2 reduction) and used Cu/UiO-66 (after plasma reaction) at 6.4 K (left); Second integrals of EPR spectra (right). The small peak at g = 2.0 in spectra was identified as emanating from the cavity of the EPR instrument in this particular experiment; the signal was not from the sample.

High-angle annular dark-field scanning transmission electron microscopy (HAADF-STEM) images and Energy dispersive X-ray spectroscopy (EDX) mapping of Cu/UiO-66

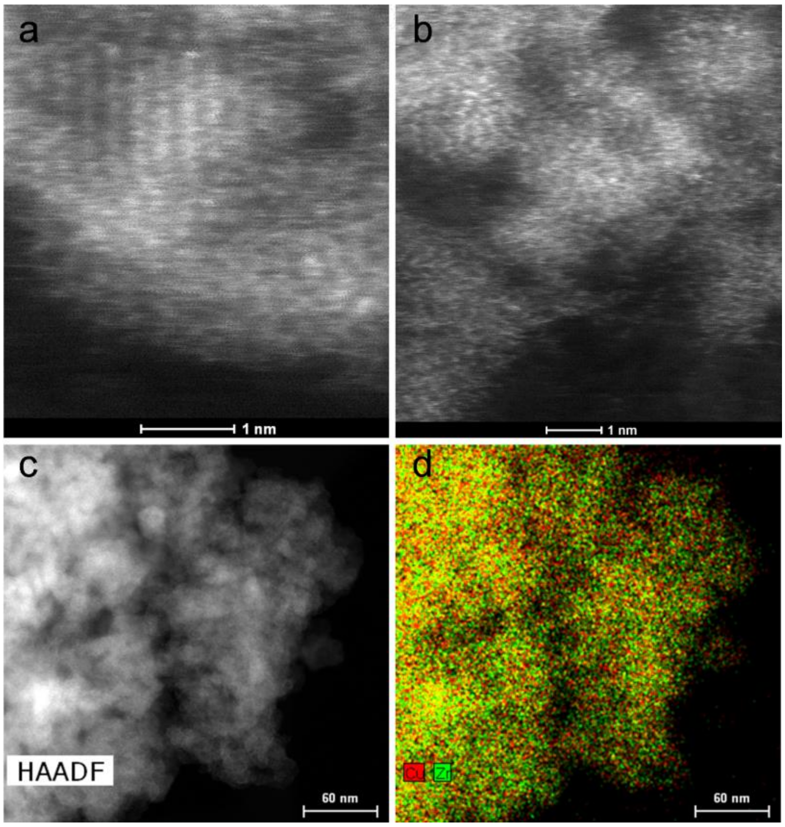

Figure S9. High resolution HAADF-STEM images of (a) fresh Cu/UiO-66 (after H_2 reduction) and (b) used Cu/UiO-66 (after plasma reaction). (c, d) HAADF-STEM image and EDX mapping of fresh Cu/UiO-66 (after H_2 reduction).

Ultraviolet-visible (UV-Vis) spectra

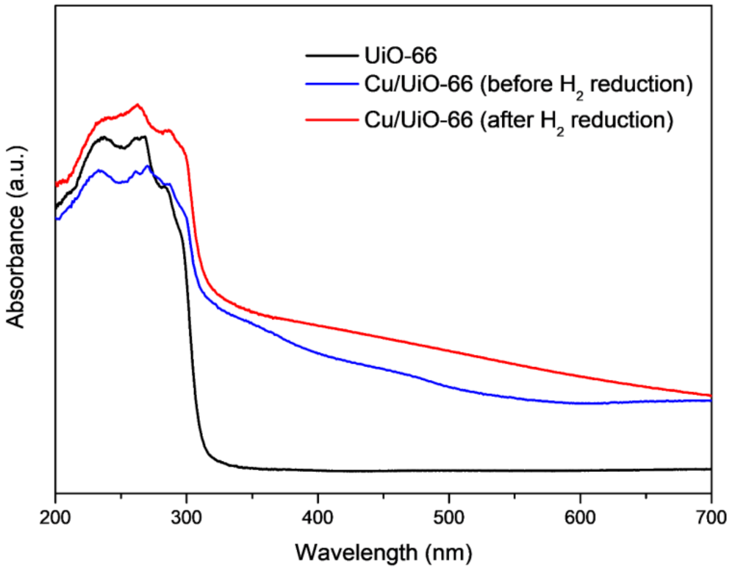

Figure S10. UV-Vis spectra of UiO-66, Cu/UiO-66 before and after H_2 reduction.

Deconvolution of ^{1}H MAS NMR spectra

It is worth noting that there are noticeable correlation peaks between ^{1}H from the ligand and adsorbed solvent ^{1}H (at $\delta_{DQ}\{^{1}H\}$~11 ppm in Figure 2d in the main text), consistent with the ^{13}C MAS NMR spectrum of reduced UiO-66 (Figure 2a in the main text). This confirms the presence of a small amount of adsorbed solvent (DMF). Formate anions are not detected in this study, which has also previously been the case for UiO-66 prepared using a similar synthesis[1].

Pristine UiO-66 displays a variety of hydrogen environments (Figure 2b in the main text), including a large narrow peak at $\delta\{^{1}H\}$~0.4 ppm that is not observed in dehydroxylated UiO-66 (H_2-reduced UiO-66). This peak accounts for more relative intensity (~0.3) compared with ^{1}H from the BDC^{2-} ligand than would be expected for μ^3–OH species of a fully hydroxylated $\{Zr_6O_4(OH)_4\}$ cluster (~0.2), indicating the presence of additional hydroxide anions[17] or water molecules that are hydrogen bonded to these μ^3–OH species. Further evidence for this can be taken from the two-dimensional (2D) double-quantum single-quantum (DQ-SQ) ^{1}H homonuclear dipolar correlation NMR spectrum of pristine UiO-66 (Figure 2d in the main text), where no correlation peaks are detected for the μ^3–OH species. This suggests that the protons from these species are in fast exchange compared with the timescale of the NMR experiment[18].

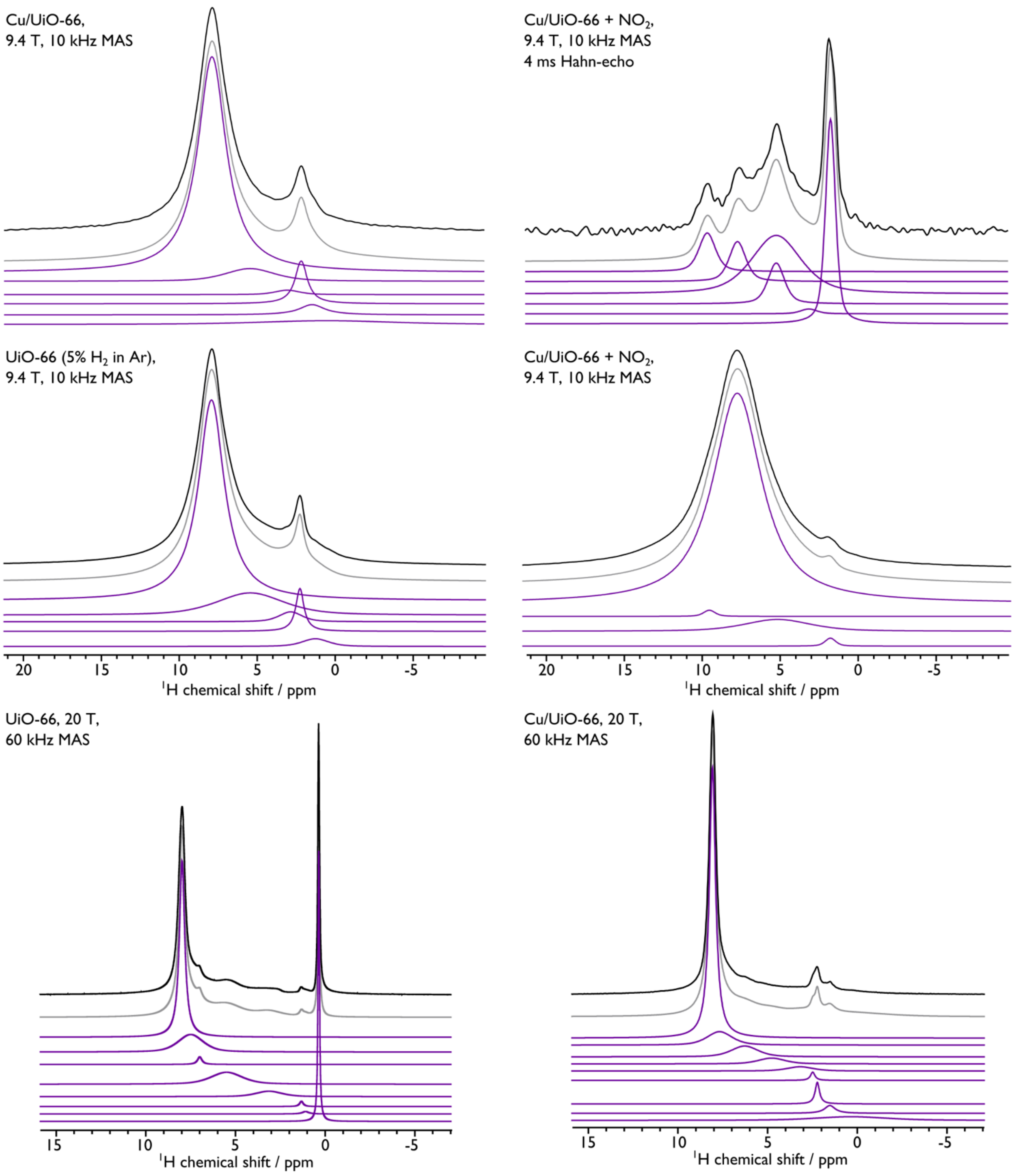

Figure S11. Non-definitive deconvolution of ^{1}H MAS NMR spectra. ^{1}H DEPTH (unless indicated) and ^{1}H-Hahn-echo (as indicated) experiments were recorded at 9.4 T or 20.0 T using a MAS frequency of 10 kHz or 60 kHz of various catalyst preparations (as indicated). Experimental spectra (black), deconvolution (purple) and summation of the deconvoluted peaks (grey).

Table S3. Integral intensities from fits to ^{1}H DEPTH NMR spectra (from Figure S11).

Catalyst (field)	^{1}H chemical shift / ppm	Linewidth / Hz	Relative intensity (±0.5 to 20 BDC ^{1}H)
UiO-66 (20 T)	8.0	300	14.4
	7.5	1400	5.6
	7.0	250	0.5
	5.5	1750	4.7
	3.1	1450	1.8
	1.3	200	0.3
	1.1	350	0.2
	0.4	80	5.7
	8.0	800	20.0
H_2-reduced UiO-66 (9 T)	5.5	2000	4.7
	2.9	700	0.7
	2.3	250	1.3
	1.3	800	0.7
Cu/UiO-66 (9 T)	8.0	880	20.0
	5.6	1500	1.7
	3.2	750	0.3
	2.3	380	1.7
	1.6	780	0.9
	0.4	4000	1.3
Cu/UiO-66 (20 T)	8.0	350	17.0
	7.7	1400	3.0
	6.3	1500	2.5
	4.8	1500	1.3
	3.2	1450	0.9
	2.4	250	0.4
	2.2	250	1.0
	1.5	600	0.9
	0.4	4000	2.2

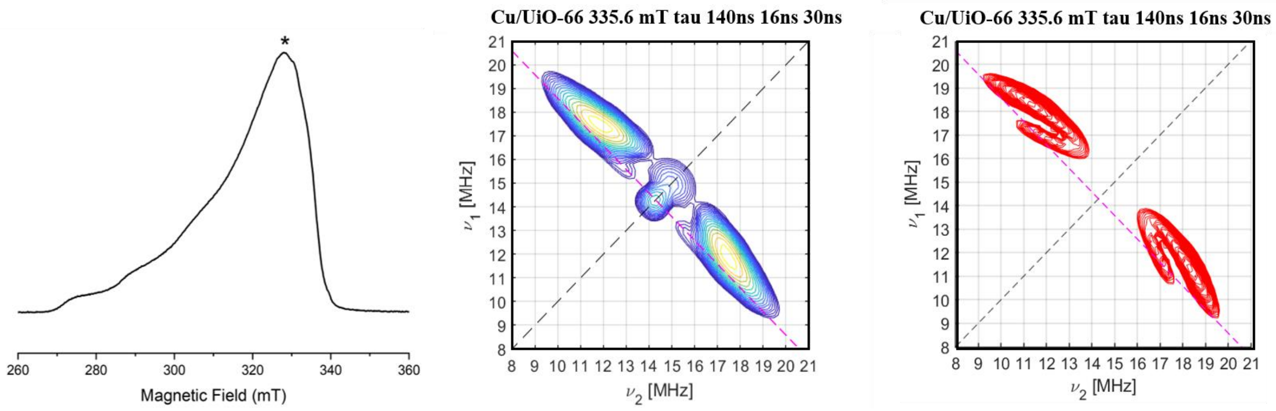

Figure S12. Left: echo-detected (ED) EPR spectrum of Cu/UiO-66 (after H_2 reduction). Middle: X-band (9.7368) ^{1}H HYSCORE spectrum measured at static magnetic field of 335.6 mT (recorded at 7 K). Right: simulated spectrum with parameters in Table S4.

Table S4. Simulation parameters of Pulse ^{1}H HYSCORE EPR spectra at X-band of Cu/UiO-66 (after H_2 reduction) shown in Figure S12.

Cu/UiO-66							
Nuclei	Electron Spin	Nuclei Spin	g-tensor	Euler angle (degree)	Aiso (MHz)	T (MHz)	Distance (Å)
^{1}H	1/2	1/2	[2.074 2.074 2.345]	[0 90 0]	-1	6.2	2.3

The local environment of the Cu(II) site was investigated by hyperfine sublevel correlation (HYSCORE) spectroscopy[4], revealing hyperfine interactions with surrounding ^{1}H nuclei (Figure S12). The spectra were modelled considering contributions from the electron-nuclear (^{1}H) dipolar and isotropic hyperfine interactions: $A = A_{dip} + A_{iso}$. The dipolar ^{1}H hyperfine interaction matrix is defined with the local z-axis directed along the Cu(II)–H vector and calculated in the high-field approximation as $A_{dip} = [-T;-T;+2T]$. Here, $T = \mu_0 g_e g_n \mu_e \mu_n / 4\pi r^3$, where μ_0 is the vacuum permeability, μ_n is the nuclear magneton, g_n is the nuclear g-factor and r is the Cu···H distance. Transformation of the $\boldsymbol{A}$ matrix into the molecular frame, defined by the g-tensor frame of the Cu(II) site (X||g_{xx}, Y||g_{yy}, Z||g_{zz}), used a single Euler angle corresponding to a rotation about the A_y axis, i.e. rotating the Cu-H vector into the g_{xz} plane.

The ^{1}H HYSCORE signal of Cu/UiO-66 reveals a pair of ridges shifted away from the anti-diagonal in the (+,+) quadrant. The shape and extent of these ridges are characteristic of protons from H_2O coordinated in the equatorial (i.e. $g_{x,y}$) plane of a Cu(II) ion[19-22]. A good simulation is achieved with the hyperfine parameters $A_{iso} = -1$ MHz, $T = +6.2$ MHz where we fixed the Euler angles [α, β, γ] = [0, 90, 0]. There is some uncertainty

in the actual value of β, but it must be nearer in-plane than out-of-plane. Distributions of A_{iso}, T and β (which are likely in these disordered materials) will lead to broadening of the HYSCORE ridges. There is also evidence of a second, more weakly coupled ^1H site giving rise to intensity along the anti-diagonal, and this introduces some further uncertainty in the parameters. Nevertheless, in the point-dipole approximation, the value of T corresponds to a distance of ~2.3 Å, consistent with a Cu–O–H connectivity. The main proton ridges are not present in the HYSCORE spectrum of the sample after dehydration in vacuum, confirming that they arise from coordinated water. Thus, the ^1H HYSCORE data are consistent with the coordination modes of Cu sites as {Zr–O(H)–Cu(OH_2)–O(H)–Zr} and {Zr–O–Cu(OH_2)–O–Zr}, as described in the main text, where the g_{zz} axis would be perpendicular to the coordination plane of the Cu(II) ion based on simple crystal field arguments.

2. Experimental setup for catalytic testing

The catalytic performance, including NO_2 conversion and N_2 selectivity, over the prepared catalysts were tested under non-thermal plasma (NTP) activated conditions. The gas flow rate of NO_2 was 100 mL min^{-1} at 25 °C (500 ppm, diluted in helium) controlled by mass flow controllers. The reaction temperature was controlled using a fan and monitored by an infrared thermometer (IRT670, General Tools & Instruments). The same gas feed was used to test the catalytic performance of all the catalysts in this work. Prior to the NTP-assisted catalytic reaction, all catalysts were packed in a fixed-bed reactor[23] and pre-treated at 80 °C for 1 h under a flow of helium (100 mL min^{-1}) to remove the residual water in the system. A gas mixture of NO_2 and He was then allowed to pass through the fixed-bed reactor to test the catalytic performance of each catalyst.

For the NTP-assisted reaction, the reactor comprised of two coaxial quartz tubes; the outside diameter of the outer tube was 6 mm and the outside diameter of the inner tube was 3 mm giving a discharge gap of 0.5 mm[23]. The outer tube was covered by a metal mesh electrode that was connected to a high-voltage output, and a metal wire electrode (ground electrode) was placed inside the inner tube. The catalysts (MOF powders) were packed in the discharge region to ensure that plasma was generated around the catalysts. An alternating current plasma generator (CPT-2000K, 0-25 kV, 10 kHz) was used to ignite the plasma, and an oscilloscope (Tektronix TDS 2022B) was used to monitor the electrical parameters. The discharge power used for reaction was about 0.6 W and the specific energy input (SEI) was calculated to be 0.4 kJ L^{-1}, with the AC peak-to-peak voltage (Vpk-pk) at around 13±0.5 kV and a frequency of 10 kHz.

The gaseous products were detected using (i) a Bruker Matrix MG5 FTIR spectrometer (resolution = 0.5 cm^{-1}) for NO_x (NO_2, NO and N_2O) and (ii) a mass spectrometer (Hiden QGA quantitative gas analysis system, Hiden Analytical Ltd.) for N_2 and O_2. The calculation of NO_2 conversion and N_2 (NO, N_2O) selectivity (S) is described in equations (1) to (4).

$$C_{\mathrm{NO_2}} = \frac{[\mathrm{NO_2}]_{\mathrm{inlet}} - [\mathrm{NO_2}]_{\mathrm{outlet}}}{[\mathrm{NO_2}]_{\mathrm{inlet}}} \times 100\ \% \qquad (1)$$

$$S_{\mathrm{N_2}} = \frac{[\mathrm{NO_2}]_{\mathrm{inlet}} - [\mathrm{NO_2}]_{\mathrm{outlet}} - [\mathrm{NO}]_{\mathrm{outlet}} - 2[\mathrm{N_2O}]_{\mathrm{outlet}}}{[\mathrm{NO_2}]_{\mathrm{inlet}} - [\mathrm{NO_2}]_{\mathrm{outlet}}} \times 100\ \% \qquad (2)$$

$$S_{\mathrm{NO}} = \frac{[\mathrm{NO_2}]_{\mathrm{inlet}} - [\mathrm{NO_2}]_{\mathrm{outlet}} - 2[\mathrm{N_2}]_{\mathrm{outlet}} - 2[\mathrm{N_2O}]_{\mathrm{outlet}}}{[\mathrm{NO_2}]_{\mathrm{inlet}} - [\mathrm{NO_2}]_{\mathrm{outlet}}} \times 100\ \% \qquad (3)$$

$$S_{\mathrm{N_2O}} = \frac{[\mathrm{NO_2}]_{\mathrm{inlet}} - [\mathrm{NO_2}]_{\mathrm{outlet}} - [\mathrm{NO}]_{\mathrm{outlet}} - 2[\mathrm{N_2}]_{\mathrm{outlet}}}{[\mathrm{NO_2}]_{\mathrm{inlet}} - [\mathrm{NO_2}]_{\mathrm{outlet}}} \times 100\ \% \qquad (4)$$

3. Characterisation of used catalysts

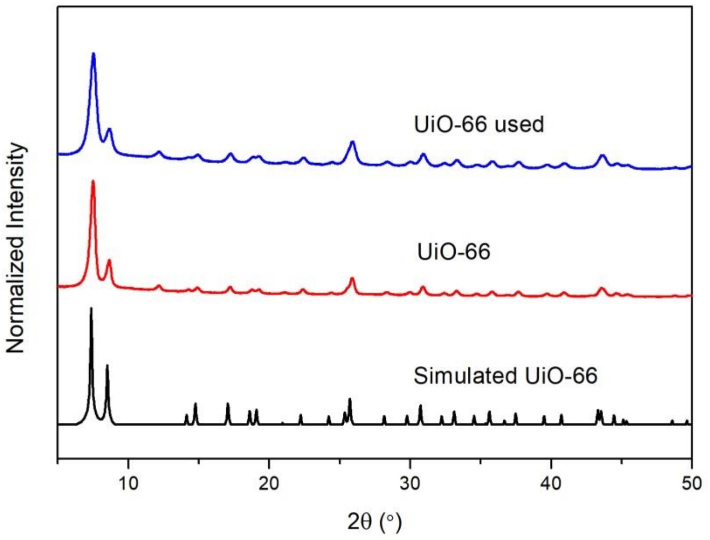

Figure S13. PXRD patterns of simulated UiO-66, fresh UiO-66 and used UiO-66.

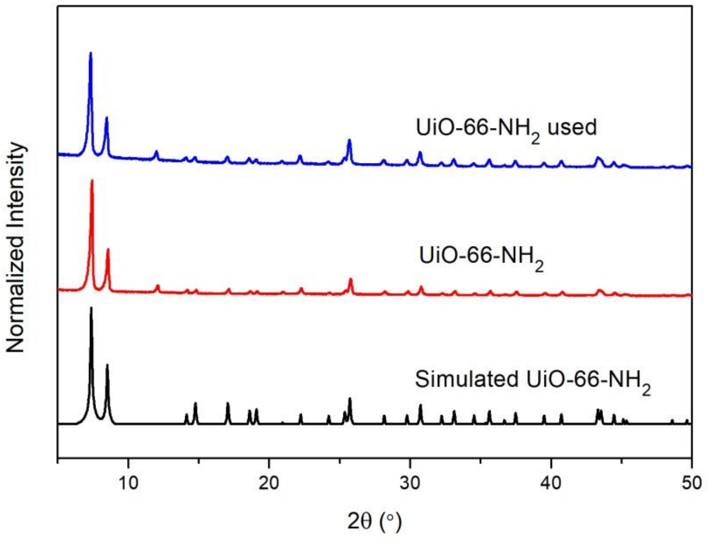

Figure S14. PXRD patterns of simulated UiO-66-NH_2, fresh and used UiO-66-NH_2.

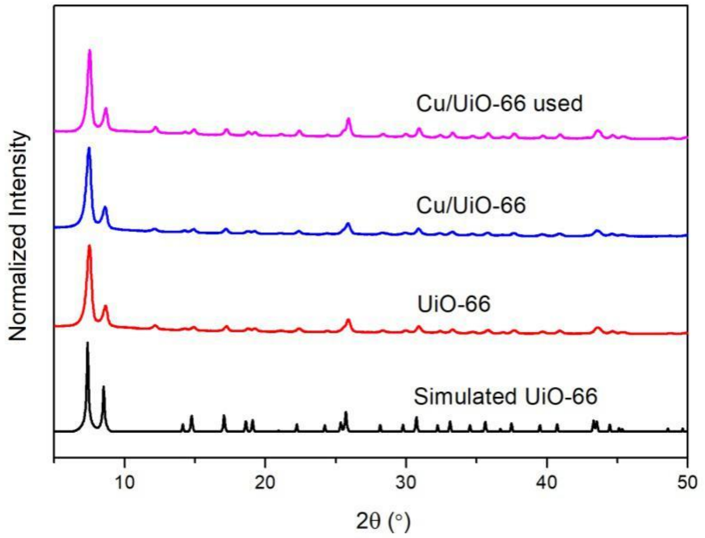

Figure S15. PXRD patterns of simulated UiO-66, as-synthesised UiO-66, fresh and used Cu/UiO-66.

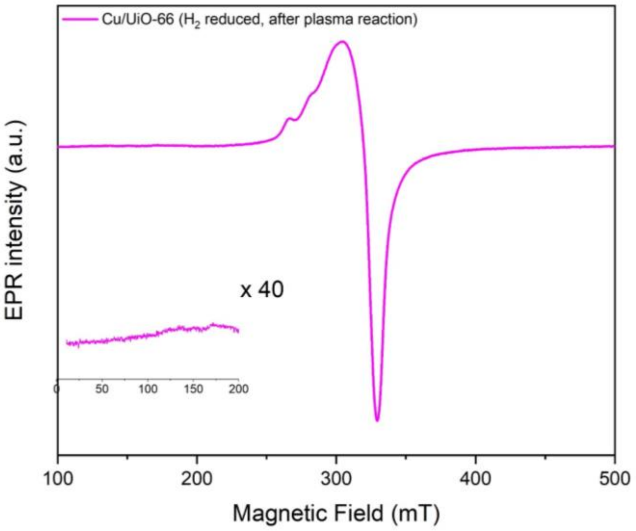

Figure S16. X-band (9.4 GHz) EPR spectra of plasma-used Cu/UiO-66 at 6.4 K. Inset: expansion of low field region, revealing absence of characteristic signals for binuclear or aggregated Cu species (S ≥ 1).

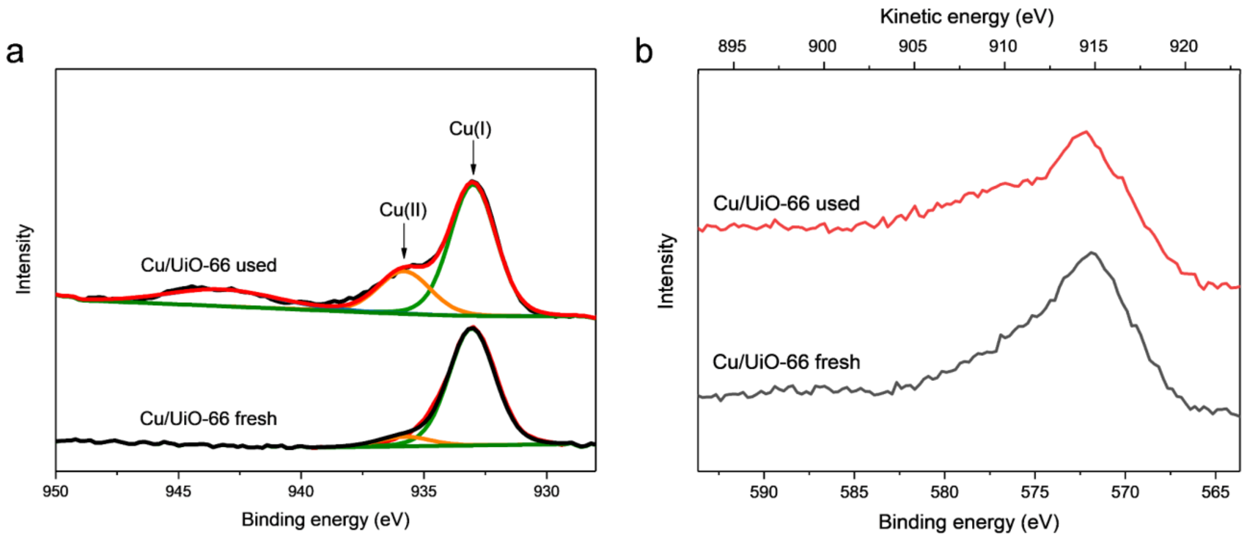

Figure S17. (a). X-ray photoelectron spectroscopy (XPS) spectra of fresh Cu/UiO-66 (after H_2 reduction) and used Cu/UiO-66 (after plasma reaction) in the Cu 2p region. (b). Cu LMM Auger spectra of fresh Cu/UiO-66 (after H_2 reduction) and used Cu/UiO-66 (after plasma reaction). Cu LMM auger and Cu 2p spectra are used to calculate modified Auger parameter of 1847.4 eV, which, with use of Wagner graphs[24,25] clearly indicates Cu(I) as the main Cu species in the catalyst. (Modified Auger parameter = position of Cu 2p 3/2 in binding energy + position of maximum of Cu LMM auger line in kinetic energy)

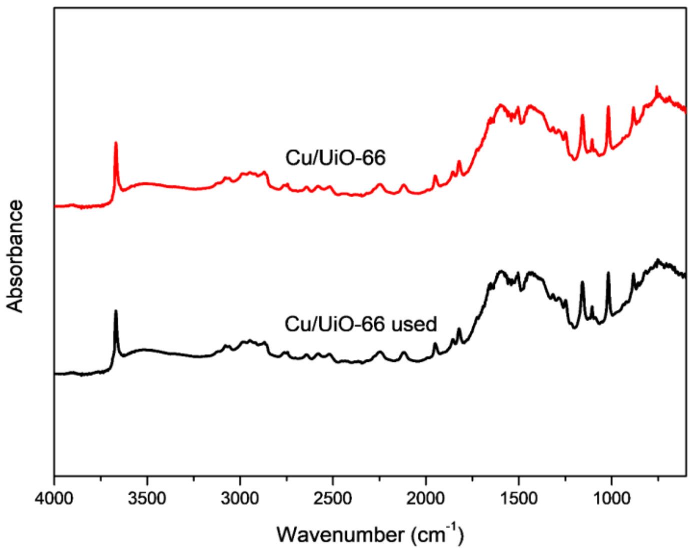

Figure S18. DRIFTS spectra of fresh (red) and used (black) Cu/UiO-66. The spectrum of KBr has been subtracted as the background.

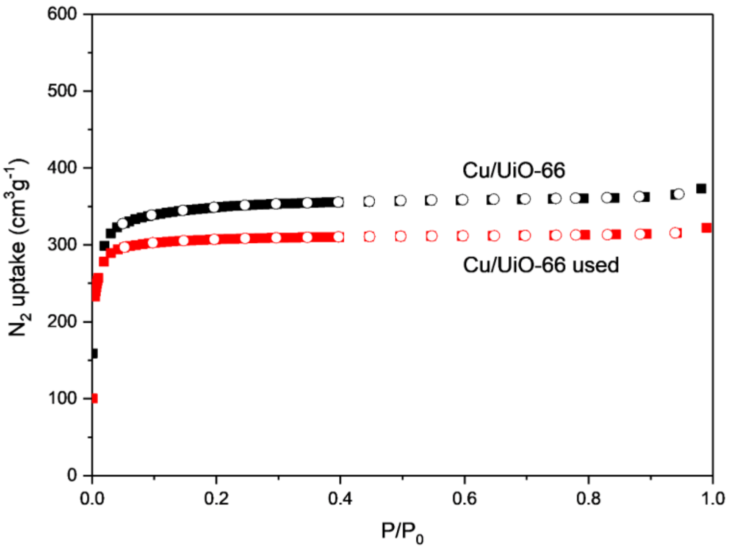

Figure S19. Nitrogen adsorption isotherms of fresh (black) and used (red) Cu/UiO-66.

Table S5. Summary of BET surface areas of fresh and used Cu/UiO-66.

Catalyst	Calculated BET surface area (m^2 g^{-1})
Cu/UiO-66 fresh	1068
Cu/UiO-66 used	963

4. Studies of the reaction mechanism

4.1 *In situ* and *operando* DRIFTS spectra

DRIFTS measurements were carried out using a Bruker Vertex 70 FTIR spectrometer. The *in situ* DRIFTS experiments of the MOF-based catalysts were conducted as a function of NO_2 adsorption. In each measurement, the MOF sample was activated at 120 °C for 2 h to remove adsorbed water, and then cooled to room temperature for spectra to be recorded of the activated samples; these were used as the background reference for analysis of the spectra of NO_2-dosed samples. For NO_2 dosing, 500 ppm of NO_2 (diluted in helium, 50 mL min^{-1}) was introduced into the DRIFTS cell for 2 h and spectra recorded. The NO_2 gas stream was replaced by pure helium (50 mL min^{-1}) to purge the DRIFTS cell for 30 mins to remove the weakly adsorbed NO_2. The *operando* DRIFTS experiments of the decomposition of NO_2 over catalyst under plasma on and off conditions were carried out with a specifically designed *in situ* plug flow cell, with the plasma generated in the catalyst bed using a modified Spectra Tech Collector II DRIFTS accessory[26]. The plasma was generated using an alternating current power source (PVM500 model) and the electrical parameters were monitored using an oscilloscope (Tektronix TBS1062) that was connected to the reactor through a high voltage probe (Tektronix, P6015). The applied voltage was 8±0.5 KV at a frequency of 27 kHz. During the DRIFTS measurements, the IR spectra were recorded with a resolution of 4 cm^{-1} and with an accumulation of 128 scans for every 60 s.

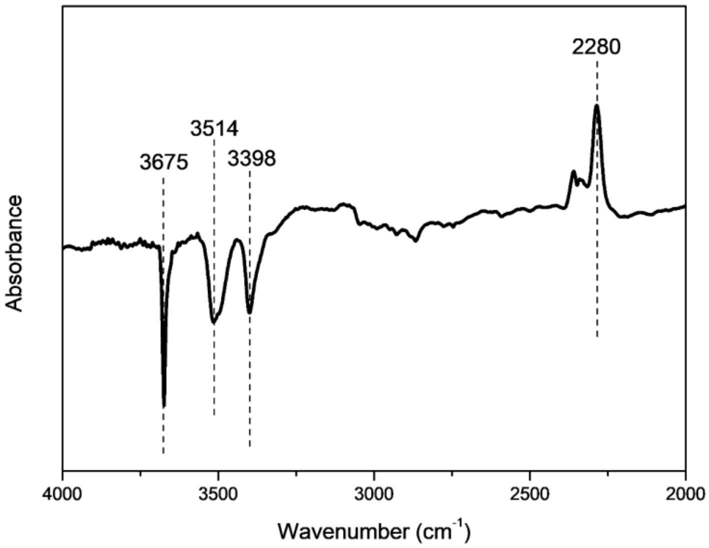

Figure S20. *Operando* DRIFTS spectrum for UiO-66-NH_2 exposed to NO_2 (500 ppm NO_2 in He) under steady-state NTP conditions. The DRIFTS spectrum was recorded at a resolution of 4 cm^{-1}. The spectrum of bare UiO-66-NH_2 has been subtracted as the background.

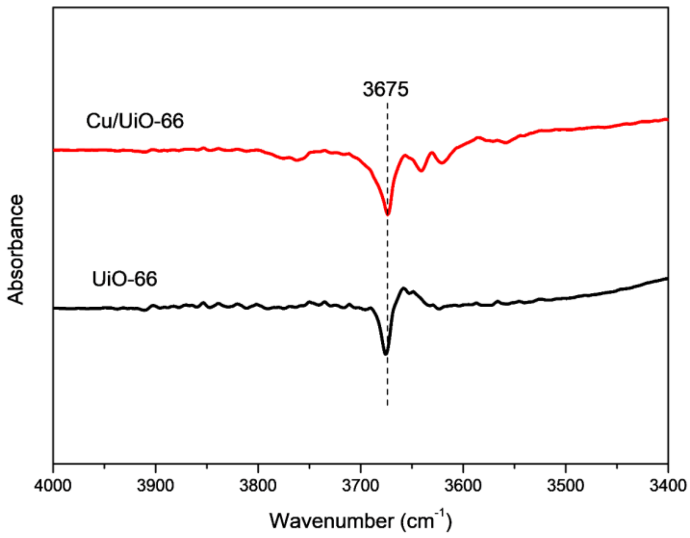

Figure S21. *In situ* DRIFTS spectra of adsorbed NO_2 on Cu/UiO-66 and UiO-66. The DRIFTS spectra were recorded at a resolution of 4 cm^{-1}. The background spectra of the bare MOF have been subtracted.

4.2 *In situ* EPR spectroscopy

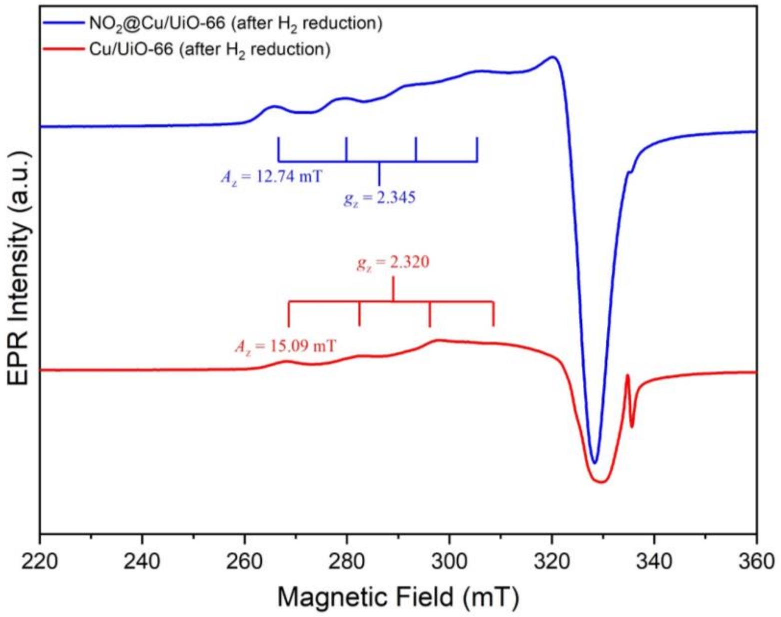

Figure S22. EPR spectra of Cu/UiO-66 after H_2 reduction (red) and Cu/UiO-66 after H_2 reduction, then NO_2 loading (blue) at 6.4 K. The spin Hamiltonian used for the simulation of the dominant contribution to the spectrum was: $H = g_z m_B S_z B_z + g_{x,y} m_B(S_x B_x + S_y B_y) + A_z S_z I_z + A_{x,y}(S_x I_x + S_y I_y)$, where g is g-tensor, S is the electron spin, I is the Cu nuclear spin, A is the $^{63/65}$Cu hyperfine interaction (HFI) and B is the applied magnetic field. The small peak at g = 2.0 in the spectra was identified as emanating from the cavity of the EPR instrument in this particular experiment; the signal is not from the sample.

Table S6. EPR simulation parameters of Cu(II) signals indicated in the spectra of Figure S22.

	treatment	A_Z (mT)	g_z	g_{xy}
Cu/UiO-66	before NO_2 loading	15.09	2.320	2.074
	after NO_2 loading	12.74	2.345	2.061

4.3 *In situ* MAS NMR spectroscopy

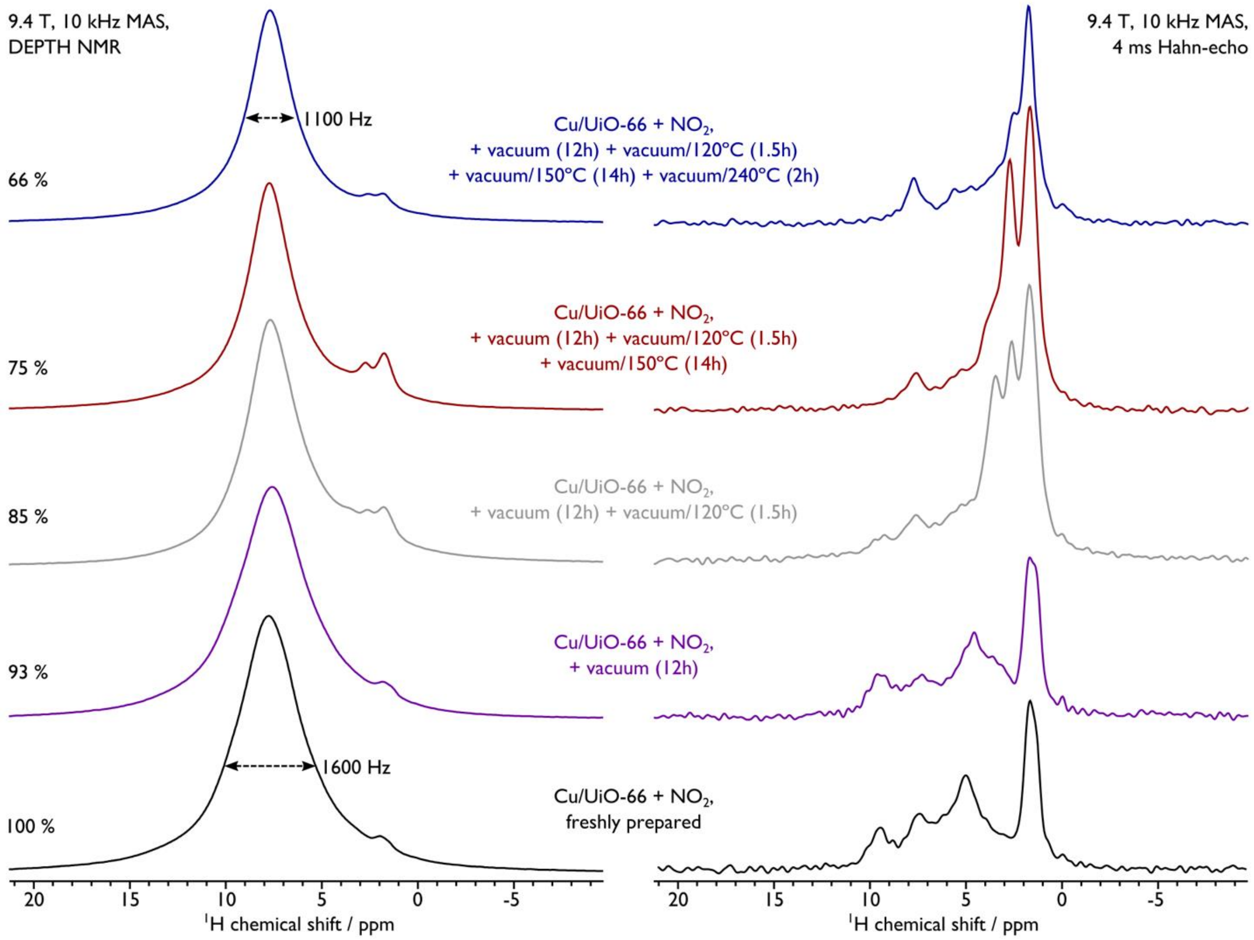

Figure S23. *In situ* ^{1}H DEPTH (left) and ^{1}H-Hahn-echo (right) MAS NMR spectra of Cu/UiO-66 dosed with NO_2 for 15 mins (black) and the same sample after various NO_2-desorption treatments (as indicated).

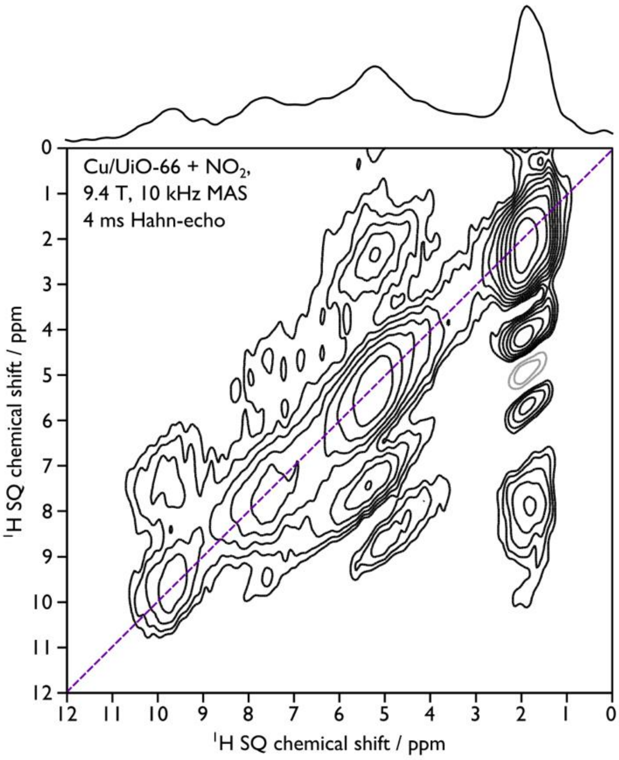

Figure S24. 2D ^{1}H MAS NMR SQ-SQ homonuclear dipolar correlation spectrum of Cu/UiO-66 dosed with NO_2 for 15 mins. RFDR[27] for 2 ms was used for the mixing period, and FSLG decoupling[28] was used to obtain improved ^{1}H resolution in the indirect dimension. A Hahn-echo (4 ms total echo time) was employed after the mixing period for spectral editing. 512 transients were acquired for each of 32 complex (STATES-TPPI) t_1 increments of 75.53 μs.

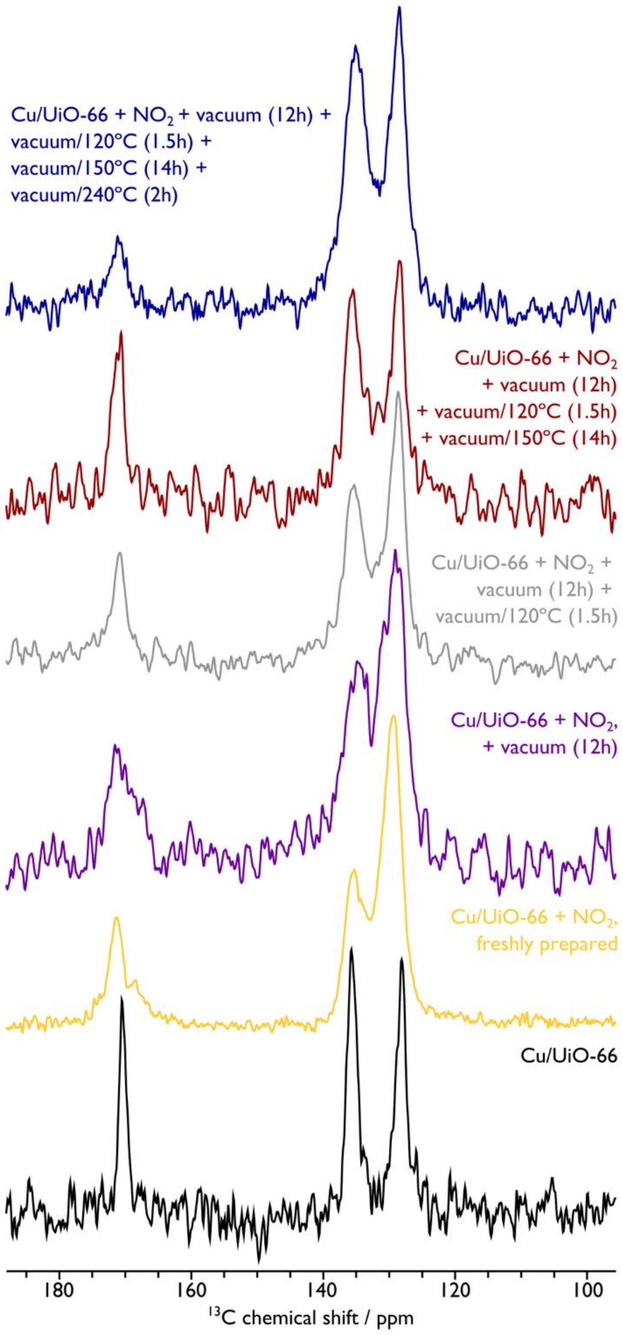

Figure S25. {^{1}H-}^{13}C CP MAS NMR spectra of UiO-66 (black), Cu/UiO-66 dosed with NO_2 for 15 mins (yellow) and the same sample after various NO_2-desorption treatments (as indicated).

5. References

1. Abdel-Mageed, A. M. et al. Highly active and stable single-atom Cu catalysts supported by a metal–organic framework. *J. Am. Chem. Soc.* **2019**, 141, 5201–5210.

2. Chapon, L. C. Wish: The new powder and single crystal magnetic diffractometer on the second target station. *Neutron News* **2011**, 22, 22–25.

3. Parker, S. F.; Fernandez-Alonso, F.; Ramirez-Cuesta, A. J.; Tomkinson, J.; Rudic, S.; Pinna, R. S.; Gorini, G.; Castañon, J. F. Recent And Future Developments On TOSCA At ISIS. *J. Phys.: Conf. Ser.* **2014**, 554, 012003.

4. Höfer, P. et al. Hyperfine sublevel correlation (HYSCORE) spectroscopy: a 2D ESR investigation of the squaric acid radical. *Chem. Phys. Lett.* **1986**, 132, 279–282.

5. Stoll, S. & Schweiger, A. EasySpin, a comprehensive software package for spectral simulation and analysis in EPR. *J. Magn. Reson.* **2006**, 178, 42–55.

6. Fung, B. M.; Khitrin, A. K.; Ermolaev, K. An improved broadband decoupling sequence for liquid crystals and solids. *J. Magn. Reason.* **2000**, 142, 97–101.

7. Teymoori, G.; Pahari, B.; Stevensson, B.; Edén, M. Low-power broadband homonuclear dipolar recoupling without decoupling: double-quantum ^{13}C NMR correlations at very fast magic-angle spinning. *Chem. Phys. Lett.* **2012**, 547, 103–109.

8. Teymoori, G.; Pahari, B.; Eden, M. Low-power broadband homonuclear dipolar recoupling in MAS NMR by two-fold symmetry pulse schemes for magnetization transfers and double-quantum excitation. *J. Magn. Reason.* **2015**, 261, 205–220.

9. Hutter, J.; Iannuzzi, M.; Schiffmann, F.; VandeVondele, J. cp2k: atomistic simulations of condensed matter systems. *Wiley Interdiscip. Rev.: Comput. Mol. Sci.* **2014**, 4, 15–25.

10. Lippert, G.; Hutter, J.; Parrinello, M. A hybrid Gaussian and plane wave density functional scheme. *Mol. Phys.* **1997**, 92, 477–487.

11. Vandevondele, J.; Krack, M.; Mohamed, F.; Parrinello, M.; Chassaing, T.; Hutter, J. Quickstep: Fast and accurate density functional calculations using a mixed Gaussian and plane waves approach. *Comput. Phys. Commun.* **2005**, 167, 103–128.

12. VandeVondele, J.; Hutter, J. Gaussian basis sets for accurate calculations on molecular systems in gas and condensed phases. *J. Chem. Phys.* **2007**, 127, 114105.

13. Goedecker, S.; Teter, M.; Hutter, J. Separable dual-space Gaussian pseudopotentials. *Phys. Rev. B* **1996**, 54, 1703–1710.

14. Perdew, J. P.; Burke, K.; Ernzerhof, M. Generalized gradient approximation made simple. *Phys. Rev. Lett.* **1996**, 77, 3865–3868.

15. Grimme, S.; Antony, J.; Ehrlich, S.; Krieg, H. A consistent and accurate ab initio parametrization of density functional dispersion correction (DFT-D) for the 94 elements H-Pu. *J. Chem. Phys.* **2010**, 132, 154104.

16. Cheng, Y. Q.; Daemen, L. L.; Kolesnikov, A. I.; Ramirez-Cuesta, A. J. Simulation of inelastic neutron scattering spectra using OCLIMAX. *J. Chem. Theory Comput.* **2019**, 15, 1974−1982.

17. Trickett, C. A.; Gagnon, K. J.; Lee, S.; Gándara, F.; Bürgi, H.; Yaghi, O. M. Definitive molecular level characterization of defects in UiO-66 crystals. *Angew. Chem., Int. Ed.* **2015**, 54, 11162−11167.

18. Brown, S. P.; Schnell, I.; Brand, J. D.; Müllen, K.; Spiess, H. W. The Competing Effects of π−π Packing and Hydrogen Bonding in a Hexabenzocoronene Carboxylic Acid Derivative: A ^{1}H Solid-State MAS NMR Investigation. *Phys. Chem. Chem. Phys.* 2000, 2, 1735−1745.

19. Schosseler, P. M.; Wehrli, B.; Schweiger, A. Complexation of copper(ii) with carbonate ligands in aqueous solution: A CW and pulse EPR study. *Inorg. Chem.* **1997**, 36, 4490–4499.

20. Santangelo, M.; Medina-Molner, A.; Schweiger, A.; Mitrikas, G.; Spingler, B. Structural analysis of Cu(II) ligation to the 5'-GMP nucleotide by pulse EPR spectroscopy. *J. Biol. Inorg. Chem.* **2007**, 12, 767–775.

21. Pöppl, A.; Kevan, L. A practical strategy for determination of proton hyperfine interaction parameters in paramagnetic transition metal ion complexes by two-dimensional HYSCORE electron spin resonance spectroscopy in disordered systems. *J. Phys. Chem.* **1996**, 100, 3387–3394.

22. Maurelli, S.; Ruszak, M.; Witkowski, S.; Pietrzyk, P.; Chiesa, M.; Sojka, Z. Spectroscopic CW-EPR and HYSCORE investigations of Cu^{2+} and O_2^- species in copper doped nanoporous calcium aluminate ($12CaO{\cdot}7Al_2O_3$). *Phys. Chem. Chem. Phys.* **2010**, 12, 10933–10941.

23. Xu, S.; Han, X.; Ma, Y.; Duong, T. D.; Lin, L.; Gibson, E. K.; Sheveleva, A.; Chansai, S.; Walton, A.; Ngo, D.; Frogley, M. D.; Tang, C. C.; Tuna, F.; McInnes, E. J. L.; Catlow, C. R. A.; Hardacre, C.; Yang, S.; Schröder, M. Catalytic decomposition of NO_2 over a copper-decorated metal–organic framework by non-thermal plasma. *Cell Rep. Phys. Sci.* **2021**, 2, 100349.

24. Wagner, C. D. Auger Lines in X-Ray Photoelectron Spectrometry. *Anal. Chem.* **1972**, 44, 967–973.

25. Naumkin, A. V.; Kraut-Vass, A.; Gaarenstroom, S. W.; Powell, C. J. NIST X-ray Photoelectron Spectroscopy Database, Version 4.1 (National Institute of Standards and Technology, Gaithersburg, 2012) DOI: http://dx.doi.org/10.18434/T4T88K.

26. Stere, C.; Chansai, S.; Gholami, R.; Wangkawong, K.; Singhania, A.; Goguet, A.; Inceesungvorn, B.; Hardacre, C. A design of a fixed bed plasma DRIFTS cell for studying the NTP-assisted heterogeneously catalysed reactions. *Catal. Sci. Technol.* **2020**, 10, 1458–1466.

27. Bennett, A. E.; Griffin, R. G. Chemical shift correlation spectroscopy in rotating solids: radio frequency-driven dipolar recoupling and longitudinal exchange. *J. Chem. Phys.* **1992**, 96, 8624–8627.

28. Bielecki, A.; Kolbert, A. C.; Levitt, M. H. Frequency-switched pulse sequences: Homonuclear decoupling and dilute spin NMR in solids. *Chem. Phys. Lett.* **1989**, 155, 341–346.
